# A Therapeutically Active Minibody Exhibits an Antiviral Activity in Oseltamivir-Resistant Influenza-Infected Mice via Direct Hydrolysis of Viral RNAs

**DOI:** 10.3390/v14051105

**Published:** 2022-05-21

**Authors:** Yongjun Lee, Phuong Thi Hoang, Dongjun Kim, Ramadhani Qurrota Ayun, Quynh Xuan Thi Luong, Kyungho Na, Taehyun Kim, Yeonsu Oh, Won-Keun Kim, Sukchan Lee

**Affiliations:** 1 Department of Integrative Biotechnology, Sungkyunkwan University, Suwon 16419, Korea; 88yjl11@naver.com (Y.L.); hoangphuong06cs@gmail.com (P.T.H.); rlaehdwns1535@gmail.com (D.K.); ramadhani.qurrota@gmail.com (R.Q.A.); quynh.ltx2017@gmail.com (Q.X.T.L.); superman13@g.skku (K.N.); 2 Novelgen Co., Ltd., R&D Center, 77, Changnyong-daero 256 Beon-gil, Yeongtong-gu, Suwon 16229, Korea; thkim@novelgen.co.kr; 3 College of Veterinary Medicine and Institute of Veterinary Science, Kangwon National University, Chuncheon 24341, Korea; yeonoh@kangwon.ac.kr; 4 Department of Microbiology, College of Medicine, Hallym University, Chuncheon 24252, Korea; wkkim1061@gmail.com; 5 Institute of Medical Science, College of Medicine, Hallym University, Chuncheon 24252, Korea

**Keywords:** H1N1/H275Y, 3D8 scFv, antivirus, prophylactic effect, therapeutic effect

## Abstract

Emerging Oseltamivir-resistant influenza strains pose a critical public health threat due to antigenic shifts and drifts. We report an innovative strategy for controlling influenza A infections by use of a novel minibody of the 3D8 single chain variable fragment (scFv) showing intrinsic viral RNA hydrolyzing activity, cell penetration activity, and epidermal cell penetration ability. In this study, we examined 3D8 scFv’s antiviral activity in vitro on three different H1N1 influenza strains, one Oseltamivir-resistant (A/Korea/2785/2009pdm) strain, and two Oseltamivir-sensitive (A/PuertoRico/8/1934 and A/X-31) strains. Interestingly, the 3D8 scFv directly digested viral RNAs in the ribonucleoprotein complex. scFv’s reduction of influenza viral RNA including viral genomic RNA, complementary RNA, and messenger RNA during influenza A infection cycles indicated that this minibody targets all types of viral RNAs during the early, intermediate, and late stages of the virus’s life cycle. Moreover, we further addressed the antiviral effects of 3D8 scFv to investigate in vivo clinical outcomes of influenza-infected mice. Using both prophylactic and therapeutic treatments of intranasal administered 3D8 scFv, we found that Oseltamivir-resistant H1N1-infected mice showed 90% (prophylactic effects) and 40% (therapeutic effects) increased survival rates, respectively, compared to the control group. The pathological signs of influenza A in the lung tissues, and quantitative analyses of the virus proliferations supported the antiviral activity of the 3D8 single chain variable fragment. Taken together, these results demonstrate that 3D8 scFv has antiviral therapeutic potentials against a wide range of influenza A viruses via the direct viral RNA hydrolyzing activity.

## 1. Introduction

Influenza, commonly known as “the flu,” is an infectious pyrogenic respiratory disease caused by an influenza virus. Global outbreaks have significant health and socioeconomic impacts on their affected communities [1,2,3]. There are two main types of influenza virus: Types A and B, which routinely spread in humans and are responsible for annual seasonal flu epidemics. Influenza outbreaks result in about 3 to 5 million cases of severe illness and about 290,000 to 650,000 deaths each year according to the World Health Organization (WHO). Annual vaccinations against influenza are recommended by the WHO for those at high risk, and the vaccine is usually effective against three or four types of influenza. However, a vaccine made for one year may not be useful for the following year owing to the rapid antigenic mutation of the influenza virus [4,5].

The influenza A (H1N1/pdm09) virus (IAV), which emerged in Mexico and the United States in April 2009, caused the global influenza pandemic. The burden of disease of IAV H1N1/pdm09 continued for the next 10 years. The Centers for Disease Control and Prevention (CDC) estimated that 10.5 million people were infected and 75,000 people died from the IAV H1N1/pdm09 infection [6,7,8].

IAV is a negative-strand RNA virus with eight RNA segments. Each of the viral genomic RNAs (vRNAs) are packaged in an individual viral ribonucleoprotein (vRNP) complex. The vRNP is comprised of a vRNA, a heterotrimeric viral polymerase (e.g., PA, PB1, and PB2), and numerous copies of the viral nucleoprotein (NP) [9,10]. Unlike common RNA viruses, replication of the influenza virus takes place in the cell nucleus. The virus replication cycle is initiated by the binding of hemagglutinin (HA) protein to its receptors, allowing the virus to enter the host cell by clathrin-mediated endocytosis. The vRNPs are released into the cytosol and transported to the host nucleus. In the nucleus, vRNPs serve as templates for transcript mRNA in the synthesis of viral proteins and for complementary RNAs (cRNA) in the production of new vRNAs. Complementary RNPs (cRNPs) and viral RNPs (vRNPs) in the nucleus are formed from newly synthesized PB1, PB2, and PA NPs, and are then exported to the cytosol to be selectively assembled into budding virions [11,12].

There are many antiviral drugs against influenza viruses that target different sites in the virus cycle [13]. Most of the drugs’ targets are viral proteins that are involved in the virus cycles, such as M2 ion channel blockers (Rimantadine and Amantadine) and neuraminidase (NA) inhibitors (Oseltamivir and Zanamivir). However, viruses are emerging and evolving with the resistance to available antiviral drugs [13,14,15,16,17]. The IAV H1N1/pdm09 has gained resistance to Oseltamivir through a particular genetic change known as the “H275Y” mutation [18,19,20,21,22].

3D8 single chain variable fragment (scFv) is a recombinant single chain antibody (~28 kDa) developed by linking the variable heavy and variable light chain domains with a flexible peptide linker. The origin of 3D8 scFv was found in MRL-*lpr/lpr* mice that developed an autoimmune syndrome similar to human systemic lupus erythematosus [23,24]. 3D8 scFv has been known to penetrate various cell types through caveolae-mediated endocytosis without any carrier [25]. Moreover, 3D8 scFv has intrinsic nucleic acid hydrolyzing activity in a sequence independent manner [26]. Many previous studies have identified 3D8 scFv antiviral effect against IAV, the classic swine fever virus, human herpes simplex virus, pseudorabies virus, and multiple corona viruses [26,27,28,29,30]. While other flu drugs directed to the protein level (e.g, Oseltamivir) were ineffective on the drug resistant strains, alternative antiviral drugs have been developed to replace Oseltamivir. Meanwhile, by hydrolyzing viral RNAs directly, 3D8 scFv has emerged as a promising candidate for antiviral drugs. 3D8 scFv is able to break down viral RNA in the cytoplasm regardless of the type of viruses and perhaps even regardless of virus mutations. In previous studies, 3D8 scFv has shown antiviral activity against different viruses, but the antiviral action of 3D8 scFv has not yet been thoroughly investigated. As an RNA virus, the influenza virus has three different viral RNAs in the nucleus or cytoplasm: viral (v), complementary (c), and messenger (m) RNA [31,32]. Therefore, to understand the mechanism of action of 3D8 scFv, the target IAV RNA of 3D8 scFv in virus-infected cells should be identified throughout the virus’ entire life cycle, from its entry to its exit, in the 3D8 scFv treatment.

In this study, we demonstrated that 3D8 scFv exhibited an antiviral effect, not only on several IAVs but also on the Oseltamivir-resistant influenza A/Korean/2785/2009pdm (H1N1/H275Y) virus in both in vitro and in vivo IAV-challenged models. Furthermore, we provided insight into 3D8 scFv’s mechanism of action in terms of which influenza RNA(s) it targets. Our results indicate 3D8 scFv may serve as a novel antiviral therapeutic against IAV wild-type viruses, Oseltamivir-resistant IAV viruses, and emerging new mutants of IAV.

## 2. Materials and Methods

### 2.1. Cell Lines and Viruses

MDCK cells were maintained in Eagle’s minimal essential medium (MEM) containing 10% fetal bovine serum (Gibco, Cergy Pontois, France), 100 U/mL penicillin, and 100 μg/mL streptomycin (Hyclone, Logan, UT, USA). The cell line was purchased from the Korean Cell Line Bank and were maintained at 37 °C with 5% CO_2_. The influenza strains A/Puerto Rico/8/34 (H1N1/PR8) and A/X-31(H3N2/X-31) was kindly provided by Prof. Dae-Hyuk Kweon (Sungkyunkwan University, Korea). The pandemic H1N1/H275Y NA-mutant virus (A/Korea/2785/2009pdm: NCCP 42017; Oseltamivir-resistant) was obtained from the National Culture Collection for Pathogens. The viruses were grown in the allantoic sacs of nine-day-old embryonated eggs at 37 °C for three days. The allantoic fluid was harvested and cleared using sucrose gradient centrifugation. Viral titers were determined using plaque assays.

### 2.2. Expression and Purification of 3D8 scFv

3D8 scFv was expressed using the pIg20-3D8 plasmid-transformed *Escherichia coli* (BL21[DE3]*pLysE*) strain in Luria-Bertani broth with 100 μg/mL ampicillin and 20 μg/mL chloramphenicol at 37 °C and induced for 18 h at 26 °C by adding 1 mM isopropyl 1-thiol-β-D-galactopyranoside (IPTG). The cell culture supernatant was obtained by centrifugation at 5200× *g* for 20 min at 4 °C and was passed through a 0.22 μm filter. 3D8 scFv was purified from the supernatant using an IgG Sepharose 6 fast flow (GE Healthcare, Malborough, MA, USA) affinity column. The column was washed with ten bed volumes of PBS (pH 7.4) and then with ten bed volumes of 5 mM ammonium acetate (pH 5.0). 3D8 scFv was eluted with 0.1 M acetic acid (pH 3.4). The eluted fraction was neutralized with a 0.1 volume of 1 M Tris-HCl (pH 9.0). The concentration of purified 3D8 scFv was determined based on the extinction coefficient at 280 nm.

### 2.3. Analysis of the Antiviral Activity (Cell Viability Test) of 3D8 scFv

MDCK cells (2 × 10^4^) were cultured in 96-well plates and treated as follows: (i) 3D8 scFv pretreatment: Cells were treated with 3D8 scFv (0, 3, 5, and 7 μM) at 37 °C with 5% CO_2_ for 24 h, and then the cells were independently infected with the influenza viral strains (Multiplicity of infection (MOI) = 0.1) in serum-free media for 1 h at 37 °C. The infection media was then removed, and the cells were cultured in serum-free media (1% BSA) containing tosyl phenylalanyl chloromethyl ketone (TPCK)-treated trypsin (1 μg/mL) at 37 °C with 5% CO_2_ for 48 h; (ii) 3D8 scFv posttreatment: MDCK cells were independently infected with the three influenza virus strains (MOI = 0.1) in serum-free media for 1 h at 37 °C, followed by removal of the infection media. The medium was changed to serum-free media (1% BSA) containing 3D8 scFv (0, 3, 5, and 7 μM) and TPCK-treated trypsin (1 μg/mL) followed by incubation at 37 °C with 5% CO_2_ for 48 h (Figure S3). Then, 0.5 mg/mL of 3-[4,5-dimethylthiazol-2-yl]-2,5-diphenyltetrazolium bromide (MTT) solution was added to each well, and the cells were incubated at 37 °C with 5% CO_2_ for 4 h. The formed formazan crystals were dissolved with DMSO, and the absorbance at 595 nm was measured using an ELISA microplate reader.

### 2.4. Measurement of the Expression of Viral RNA Using qRT-PCR

Total RNA was isolated using the TRI reagent (MRC, Montgomery, OH, USA) according to the manufacturer’s instructions. The RNA concentration was determined using a spectrophotometer. cDNA was synthesized using CellScript All-in-One 5× First Strand cDNA Synthesis Master Mix (CellSafe, Yongin, Korea) according to the manufacturer’s protocol. Quantitative real-time PCR was performed using SYBR Premix Ex Taq and Rotor-Gene Q system. Data were analyzed using Rotor-Gene Q series software version 2.3.1 (Qiagen, Chadstone, VIC, Australia). The genes (*HA* and *NP*) were amplified using the indicated primers (Table S1). GAPDH was amplified as an internal control.

### 2.5. Measurement of Viral HA Expression Using Immunocytochemistry (ICC)

MDCK cells (2 × 10^4^) were cultured in eight-well chamber slides. The procedures for the 3D8 scFv treatment and virus infection (H1N1/H275Y and H1N1/PR8) were the same as described in the previous section (Figure S3). The slides were washed twice with PBS and fixed for 15 min using chilled methanol at 25 °C. Samples were permeabilized for 15 min with Intracellular Staining Perm Wash Buffer. After blocking with PBST containing 1% BSA for 1 h, the cells were incubated with the monoclonal mouse anti-3D8 scFv antibody or polyclonal rabbit anti-HA antibody (1:1000 dilution; Invitrogen) for 24 h at 4 °C. The cells were then incubated with the goat anti-mouse IgG Alexa Fluor 488 secondary antibody or goat anti-rabbit IgG TRITC secondary antibody (1:1000 dilution; Abcam), respectively, for 1 h at 25 °C. The slides were mounted using Antifade Mounting Medium with DAPI, and the cells were visualized using a Zeiss LSM 700 confocal microscope.

### 2.6. Comparison of Intracellular 3D8 scFv Retention Time by 3D8 scFv Treatment Method

MDCK cells (2 × 10^4^) were cultured in eight-well chamber slides (Nunc Lab-Tek) and treated as follows: (i) 3D8 scFv pretreatment: Cells were treated with 3 μM of 3D8 scFv at 37 °C with 5% CO_2_ for 24 h, and then the medium was changed to serum-free media (1% BSA) followed by incubation at 37 °C with 5% CO_2_. The cells were fixed after 2, 4, 6, 8, 12, and 24 h for 15 min in chilled methanol at 25 °C; (ii) 3D8 scFv posttreatment: Cells were treated with 3 μM of 3D8 scFv at 37 °C with 5% CO_2_, and then the cells were fixed after 2, 4, 6, 8, 12, and 24 h for 15 min in chilled methanol at 25 °C. The samples were permeabilized for 15 min using Intracellular Staining Permeabilization Wash Buffer (BioLegend, San Diego, CA, USA). After blocking with 1% BSA in PBST for 1 h, the samples were incubated with monoclonal mouse anti-3D8 scFv antibody (1:1000 dilution; AbClon, Seoul, Korea) at 4 °C for 24 h. The samples were incubated with goat anti-mouse IgG Alexa Fluor 488 secondary antibody (1:1000 dilution; Abcam, Cambridge, MA, USA) for 1 h at 25 °C, and the slides were mounted in Antifade Mounting Medium with DAPI (Vectashield, Burlingame, CA, USA). The samples were then visualized using a Zeiss LSM 700 confocal microscope. ImageJ software (NIH, Bethesda, MD, USA) was used for quantification of 3D8 scFv’s fluorescence signal.

### 2.7. Preparation of vRNP and Analysis of 3D8 scFv’s vRNP Hydrolyzing Activity

vRNPs were released directly from virus particles (1 × 10^5^ PFU) via treatment with 0.1% Triton X-100 in TBS buffer at 25 °C for 30 min. vRNP was incubated with 3D8 scFv purified protein (1 μg) for 1 h in TBS containing 0.1 mM MgCl_2_ at 37 °C. BSA was used as a negative control. cDNA was synthesized using CellScript All-in-One 5× First Strand cDNA Synthesis Master Mix according to the manufacturer’s protocol. The sequences corresponding to *HA, NA, M1, NP, NS1, PA, PB1*, and *PB2* were amplified with the indicated primers (Table S2), analyzed by electrophoresis on a 1% agarose gel, and stained with ethidium bromide.

### 2.8. Measurement of the Expression of Viral vRNA, cRNA, and mRNA Using qRT-PCR

The cells were harvested after 2, 4, 6, 8, 12, and 24 hpi. Total RNA was isolated using the TRI reagent (MRC, Montgomery, OH, USA) according to the manufacturer’s instructions. RNA concentration was determined using a spectrophotometer. vRNA, cRNA, and mRNA-cDNA was synthesized using M-MLV Reverse Transcriptase (Bioneer, Daejeon, Korea) according to the manufacturer’s protocol. Quantitative real-time PCR was performed using SYBR Premix Ex Taq and the Rotor-Gene Q system. Data were analyzed using Rotor-Gene Q series software version 2.3.1 (Qiagen, Chadstone, VIC, Australia). The genes (*HA* and *NP*) were amplified using the indicated primers (Tables S3 and S4). *GAPDH* was amplified as an internal control.

### 2.9. Animals and Antiviral Activity Test In Vivo

Six-week-old male specific pathogen-free (SPF) BALB/c mice (DBL, Eumseong, Korea), weighing 18–20 g, were housed under standard laboratory conditions. All animal procedures were approved by the Institutional Animal Care and Use Committee of Sungkyunkwan University (permit number: SKKUIACUC2019-03-07-3). Mice were pre/posttreated intranasally with 50 μg of 3D8 scFv for 4 d. Oseltamivir phosphate (10 mg/Kg) (Sigma-Aldrich, St. Louis, MO, USA) was administered orally for 5 d post infection. The mice were infected intranasally with a 50 μL 50% lethal dose (LD_50_) of H1N1/H275Y virus (5 × 10^4^ PFU; Figure S3A,B). After challenge with H1N1/H275Y, the mice were monitored daily for clinical signs (survival and weight loss) until day 11 post infection. Lung samples were collected for histopathological examination on 5 dpi.

### 2.10. Measurement of Viral RNA and Interferon Levels

Total RNA was extracted from lysed mouse lung tissue (5 dpi) samples using the TRI reagent according to the manufacturer’s instructions. The RNA concentration was determined using a spectrophotometer. cDNA was synthesized using CellScript All-in-One 5× First Strand cDNA Synthesis Master Mix according to the manufacturer’s protocol. The genes (*HA, NP*, and *IFN-β*) were amplified with the indicated primers (Table S1) using qRT-PCR as described previously. *GAPDH* was amplified as an internal control.

### 2.11. Comparison of Virus Titer in the Lungs

To confirm the reduction of virus titer in the lung, plaque assay was conducted following the previous study [33]. Briefly, mouse lung tissues were homogenized in 1 mL of serum-free media using TissueRuptor (Qiagen, Chadstone, VIC, Australia). The homogenate was obtained by centrifugation at 6000× *g* for 5 min at 4 °C. Confluent MDCK cells were cultured in 12-well plates and then infected with the tissue supernatant (1:1000 dilution) in serum-free media for 1 h at 37 °C followed by removal of the infection media. The medium was replaced with 1× Dulbecco’s Modified Eagle Medium (DMEM) containing TPCK-treated trypsin (1 μg/mL) and 1% agarose. The cells were further incubated at 37 °C with 5% CO_2_ for 3 d. The cells’ monolayers were fixed with 4% formaldehyde and stained with 0.5% crystal violet for visualization.

### 2.12. Immunohistochemistry (IHC)

Tissue sections were deparaffinized and rehydrated, and antigen retrieval was performed using citrate buffer at 95 °C for 20 min. Slides were washed in TBST (TBS, 0.025% Triton X-100) and blocked using a solution of 10% fetal bovine serum + 1% BSA in TBST for 2 h. The tissue sections were incubated with polyclonal rabbit anti-HA antibody (1:750 dilution) overnight at 4 °C. The tissue slides were then incubated for 1 h at 25 °C with goat anti-rabbit IgG TRITC secondary antibody (1:1000 dilution). The tissue sections were finally mounted in Antifade Mounting Medium with DAPI, and the cells were visualized using an LSM 700 Zeiss confocal microscope.

### 2.13. Morphometric Analysis

Lung tissues were collected from each group of mice. For morphometric analysis of the gross pulmonary lesion score, the method described by Harbur et al. (1995) was slightly modified [34]. Briefly, each lung lobe was assigned a number that reflected the approximate percentage of the volume of the entire lung represented by that lobe. The total points for all of the lobes formed an estimate of the percentage of the entire lung with grossly visible pneumonia.

For morphometric analysis of the microscopical pulmonary lesion score, lung tissues were fixed in 10% neutral buffered formalin. After fixation, the tissues were dehydrated through a graded series of alcohol solutions and xylene and embedded in paraffin wax. Sections (5 µm) were prepared from each tissue and stained with haematoxylin and eosin (HE). The prepared tissue slides were examined in a blinded fashion and scored for the estimated severity of the interstitial pneumonia as: 0, no lesions; 1, mild interstitial pneumonia; 2, moderate multifocal interstitial pneumonia; 3, moderate diffuse interstitial pneumonia; and 4, severe interstitial pneumonia.

For the morphometric analysis of IHC, the sections were analyzed with the NIH Image J 1.43m program (https://imagej.nih.gov/ij (accessed on 1 February 2021)) to obtain quantitative data. A total of ten fields were selected randomly, and the number of positive cells per unit area (0.25 mm^2^) was determined. The mean values were then calculated.

### 2.14. Statistical Analysis

Continuous data were analyzed with a one-way analysis of variance (ANOVA). If a one-way ANOVA was significant (*p* < 0.05), a pairwise Tukey’s adjustment was performed as a posthoc. Discrete data were analyzed by Chi-square and Fisher’s exact tests. If necessary, Pearson’s correlation coefficient and/or Spearman’s rank correlation coefficient were used to assess the data’s relationship. A value of *p* < 0.05 was considered significant. All statistical analyses were performed using the GraphPad Prism software (GraphPad Software, San Diego, CA, USA).

## 3. Results

### 3.1. 3D8 scFv Protected Cells against Oseltamivir-Resistant IAV Infection

The H1N1/H275Y-infected cells exhibited drug resistant phenotypes, but the H1N1/PR8- and H3N2/X-31-infected cells did not exhibit drug resistance (Figure S1). A549 cells were treated with different doses of 3D8 scFv (0–40 μM) for 48 h to investigate the 3D8 scFv cytotoxic dose. At 3D8 scFv concentrations up to 10 μM in the MTT assay, cell death was not observed, and with 3D8 scFv concentrations from 0 μM to 10 μM by NGS analysis, changes in differentially expressed genes (DEGs) were observed to be significant, suggesting that 3D8 scFv was not toxic and maintained its antiviral activity at the tested dose of 0–10 μM (Figure S2A,B).

**Figure 1:**
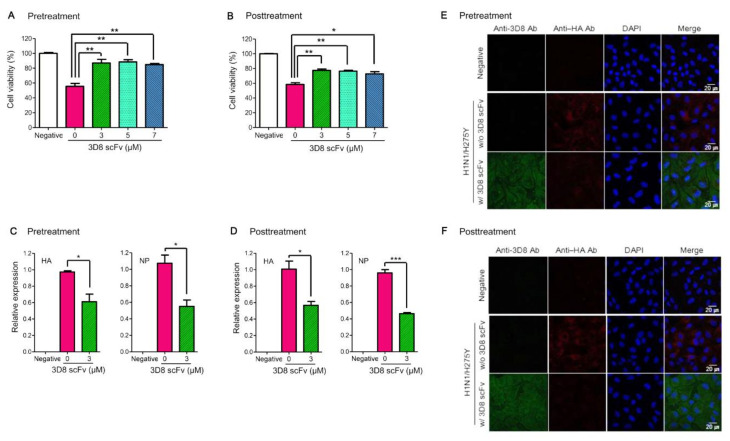
Prophylactic and therapeutic antiviral effects of 3D8 scFv against H1N1/H275Y strains in vitro. (**A**,**B**) The relative viability of 3D8 scFv-pre/posttreated MDCK cells against viruses at 48 hpi. 3D8 scFv treatment was conducted following the scheme illustrated in Figure S3. Cell viability was measured using an MTT assay. (**C**,**D**) The relative viral RNA expression (*HA* and *NP*) in 3D8 scFv (3 μM)-pre/posttreated MDCK cells against virus at 24 hpi. Data are shown as the mean ± S.E.M. of triplicate samples. Error bars indicate standard error (SE). Asterisks indicate significant differences determined by the *t*-test (* *p* < 0.05, ** *p* < 0.01, *** *p* < 0.001). (**E**,**F**) Detection of viral protein (HA) in infected MDCK cells using immunocytochemistry at 24 hpi after 3D8 scFv pre/posttreatment. Nuclei were detected using DAPI (blue), 3D8 scFv with Alexa Fluor 488 (green), and viral HA with TRITC (red). Scale bars represent 20 μm.

To investigate the antiviral effect of 3D8 scFv treatment pre- or post-viral infection, the experiment was conducted following the scheme illustrated in Figure S3. In the 3D8 scFv pretreatment group, the cell viability of H1N1/H275Y-infected cells, used as a positive control (0 μM), was initially 55.6%, and it increased by 31.5% after 3D8 scFv treatment (5 μM) (Figure 1A). Moreover, while the cell viability of H1N1/PR8-infected cells, used as a positive control, was 66.7%, cells treated with 5 μM 3D8 scFv increased their cell viability by 22% (Figure S4A). Furthermore, the cell viability of all 3D8 scFv-treated groups in the H3N2/X-31-infected cells increased by 20% (5 μM) compared with the 3D8 scFv-untreated group (54.4%; Figure S4B). In the case of 3D8 scFv posttreatment, while the cell viability of H1N1/H275Y-infected cells without 3D8 scFv treatment was 58.4%, cells treated with 3 μM 3D8 scFv showed an increase in cell viability by 19% to 77.4% (Figure 1B). While the cell viability of H1N1/PR8-infected cells (0 μM) was 63.2%, cells treated with 5 μM 3D8 scFv showed an increase of 23.7% in their cell viability (Figure S4C). Further, H3N2/X-31-infected cells treated with 3D8 scFv showed an increased cell viability from 22% up to 84.4% compared with that of cells not treated with 3D8 scFv (62.6%; Figure S4D). These results indicated that both pre- and posttreatment with 3D8 scFv of virus-infected cells increased cell viability by at least 20%.

In H1N1/H275Y-infected cells, the RNA levels of IAV *HA* and *NP* decreased to 61% and 55%, respectively, in the 3D8 scFv pretreated cells (Figure 1C). In the 3D8 scFv-posttreated cells, the RNA level of IAV *HA* and *NP* was observed decreasing by 56% and 46%, respectively (Figure 1D). H1N1/PR8- and H3N2/X-31-infected cells also showed similar reductions in viral RNA (*HA* and *NP*) levels with 45–60% (*HA*) and 52–54% (*NP*) reductions in the pretreated cells (Figure S5A,C) and 53–56% (*HA*) and 48–59% (*NP*) in the posttreated cells (Figure S5B,D).

To confirm the reduction of the virus titers, localizations of the IAV HA protein and 3D8 scFv were monitored using an immunofluorescence assay. We detected viral HA protein in the cytoplasm (red signal) of H1N1/H275Y-infected cells without 3D8 scFv treatment; however, compared with the 3D8 scFv-untreated control, the viral HA protein decreased in the cytoplasm of cells pretreated with 3D8 scFv (Figure 1E). In contrast, 3D8 scFv was localized in the cytoplasm (green signal) in all 3D8 scFv-treated cells. In the H1N1/PR8-infected cells treated with 3D8 scFv, the viral HA protein’s signals appeared to be less than those of both the positive control group and the 3D8 scFv-untreated cells (Figure S6A). Consistent with the 3D8 scFv pretreatment groups, the viral HA protein’s signals decreased in all the virus-infected and 3D8 scFv-posttreated groups compared with the infection-only group (Figure 1F and Figure S6B).

### 3.2. 3D8 scFv Hydrolyzed the vRNA of vRNP as Well as Three Types of IAV RNAs in the Cytoplasm

**Figure 2:**
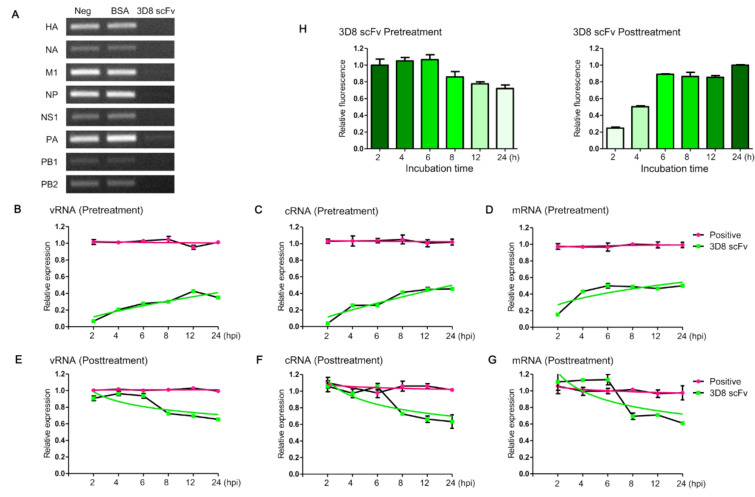
Identification of the differences in antiviral mechanisms according to 3D8 scFv treatment methods. (**A**) 3D8 scFv hydrolyzes eight viral RNAs of the vRNP complex. Total vRNP isolated from the virus particles was incubated with 3D8 scFv for 1 h at 37 °C, and then the reactions were amplified with the viral RNA specific primers. (**B**–**D**) The relative expression of vRNA, cRNA, and mRNA of viral *HA* in 3D8 scFv-pretreated MDCK cells. MDCK cells were incubated with 3D8 scFv (3 μM) in advance of virus infection (MOI = 0.1), and qRT-PCR was performed to measure the relative expression of viral *HA* RNAs. (**E**–**G**) The relative expression of vRNA, cRNA, and mRNA of viral *HA* after virus infection in 3D8 scFv-posttreated MDCK cells. qRT-PCR was performed to measure the relative expression of viral *HA* RNAs. Data are shown as the mean ± S.E.M. of triplicate samples. (**H**) Changes in the amounts of 3D8 scFv in the cytoplasm according to 3D8 scFv pre- and posttreatment, respectively.

To investigate if 3D8 scFv digested the viral RNA molecules of vRNP, the vRNPs releasing from IAV particles were incubated with 3D8 scFv, and then each RNA was amplified by RNA-specific primers. None of the viral RNAs was detected on the gel. 3D8 scFv hydrolyzed all eight vRNAs that were bound to nucleoproteins (RNPs) (Figure 2A).

To understand the antiviral effects by 3D8 scFv pre- and posttreatments, the three influenza viral RNA levels (vRNA, cRNA, and mRNA) were monitored individually using quantitative real-time PCR with *HA*- and *NP*-specific primers. In 3D8 scFv-pretreated cells, the levels of the three types of viral *HA* RNA reached 6% (vRNA), 3% (cRNA), and 15% (mRNA) compared with the positive control at 2 hpi. Over time, however, the viral RNA levels increased to 34% (vRNA), 45% (cRNA), and 49% (mRNA) and remained at these levels at 24 hpi (Figure 2B–D). Viral *NP* RNAs also showed a similar trend as seen with viral *HA* RNAs (Figure S7A–C). In 3D8 scFv-posttreated cells, all three viral *HA* RNA levels were similar to the RNA levels of the positive controls up to 6 hpi, but the RNA levels began to decrease to 72% (vRNA), 72% (cRNA), and 69% (mRNA) compared with the positive controls at 8 hpi and reached 65% (vRNA), 63% (cRNA), and 61% (mRNA) at 24 hpi (Figure 2E–G). In 3D8 scFv-posttreated cells, viral *NP* RNAs also showed a similar pattern as was observed with viral *HA* RNAs (Figure S7D–F).

To understand why antiviral effects reached similar levels at 24 hpi in both the 3D8 scFv-pre- and post-treated cells, we analyzed the changes in the amount of 3D8 scFv in the cytoplasm for up to 24 h according to the treatment methods (Figure S8). In 3D8 scFv-pretreated cells, when 3D8 scFv was exposed to the cells for 24 h in advance and then the culture media was replaced with new media without 3D8 scFv, the intensity of the intracellular Alexa488-labeled 3D8 scFv protein gradually decreased as time progressed and reached 71% at 24 h (Figure 2H). In 3D8 scFv-posttreated cells, the intensity of the 3D8 scFv signals increased from 24% at 2 h after treatment to 89% at 6 h and then reached a maximum at 24 h. In both the pre- and posttreatments, 6h was the turning point for the decrease and increase in 3D8 scFv’s localization in the cytoplasm, respectively. The amounts of 3D8 scFv localized in the cytoplasm after the 3D8scFv treatments strongly correlated with 3D8 scFv’s antiviral activity at different time points after viral infection (Figure 2B–G).

### 3.3. 3D8 scFv Increased the Survival of H1N1/H275Y-Infected Mice

To evaluate 3D8 scFv antiviral activity clinically, mice were used as an in vivo experimental model. The control mice, treated with 50 µg of 3D8 scFv for 4d, did not show any abnormal clinical signs (Figure S9A). Histopathologically, there was no abnormal change in the mice’s lung parenchyma and corresponding interstitium (Figure S9B,C). Of note, 3D8 scFv was found to be localized in the epithelial lining of the bronchioles and alveoli for 48 h after administration (Figure S9D,E).

**Figure 3:**
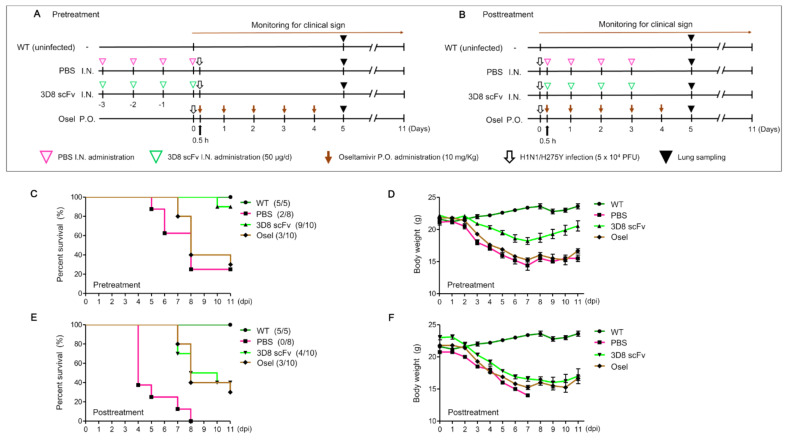
In vivo assessment of antiviral effects of 3D8 scFv against H1N1/H275Y infection. (**A**,**B**) Schematic diagram of 3D8 scFv treatment and H1N1/H275Y infection protocol. Mice were infected with H1N1/H275Y virus and pre/posttreated intranasally with 3D8 scFv (50 µg/d) for 4 d. Oseltamivir (Osel) (10 mg/Kg) was orally administered (5 d consecutively). The clinical signs in the mice were observed for 11 d. The mice were euthanized (5 dpi) for lung sampling. (**C**,**E**) The survival rates of the infected mice. (**D**,**F**) The mean body weight of the infected mice. (WT *n* = 5, PBS pre/posttreatment *n* = 8, 3D8 scFv pre/posttreatment *n* = 10, Oseltamivir administration *n* = 10).

To test the efficacy of 3D8 scFv, five groups, with 8–10 mice per group, of six-week-old male Balb/c mice were challenged intranasally with a 50% lethal dose (LD_50_, 5 × 10^4^ PFU) of H1N1/H275Y, and then they were treated with PBS (pre-/posttreatment), 3D8 scFv (pre-/posttreatment), or Oseltamivir. Clinical signs in the mice were monitored for the next 11 d, including monitoring for body weight change, coughing, a hunched back, coarse fur, gathering in clusters (fever), abnormal discharge, and survival (Figure 3A,B). The results of monitoring the survival rates revealed that 90% of the 3D8 scFv pretreatment group survived, while only 25% of the PBS-pretreatment group survived (Figure 3C). In the 3D8 scFv-posttreatment group, 40% survived, yet none of the PBS-posttreatment group survived (Figure 3E). In all, 30% of the Oseltamivir-treatment group survived.

In terms of change in body weight, in the PBS-pretreatment group, the average body weight was 21.1 g at 0 days post infection (dpi) and decreased by 27% to 15.5 g at 11 dpi (Figure 3D). In the 3D8 scFv-pretreatment group, the average body weight was 22.2 g at 0 dpi and decreased by about 18% to 18.2 g at 7 dpi; however, the average body weight rebounded to 20.6 g at 11 dpi. In the Oseltamivir group, the average body weight was 21.8 g at 0 dpi, and it decreased by 30% at 7 dpi and rebounded at 11 dpi.

In the PBS-postreatment group, the average body weight was 20.8 g at 0 dpi, but no mice in the group were alive at 8 dpi (Figure 3E). In the 3D8 scFv-posttreatment group, the average body weight was 23 g at 0 dpi, and it decreased by about 30% to 16 g at 10 dpi (Figure 3F); however, the average body weight of the 3D8 scFv–treated group started to recover at 7 dpi. Obviously, the mouse body weight in the 3D8 scFv-pretreatment group recovered faster in comparison to that of the 3D8 scFv-posttreatment group.

These results demonstrated that 3D8 scFv had a noticeable clinical benefit, which consequently enhanced the mice’s survival rate, and, although it could not prevent an initial weight loss due to the viral challenge, 3D8 scFv was also able to restore the mice’s body weight.

### 3.4. 3D8 scFv Administration Lowered Histopathological Lesions in the Lungs of Viral-Challenged Mice

**Figure 4:**
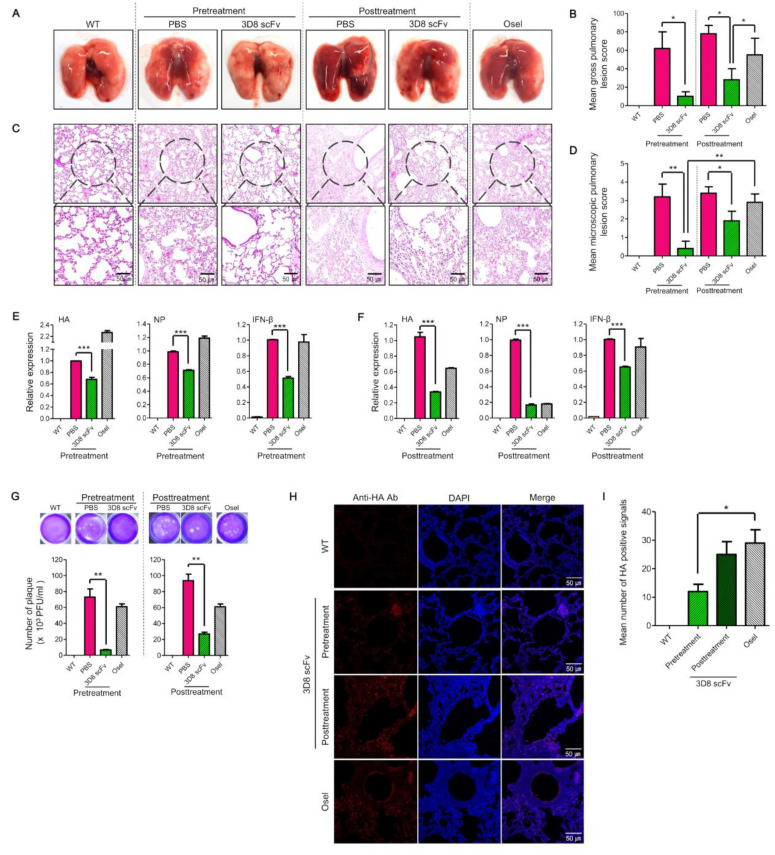
Histopathological analysis of the lung tissues of H1N1/H275Y-infected mice after intranasal 3D8 scFv treatment. (**A**,**B**) A morphological comparison and the gross pulmonary lesion scores of the mice’s lungs. The mice’s lungs were harvested at 5 dpi. (**C**,**D**) A histopathological comparison and the microscopic pulmonary lesion scores of the mice’s lung sections. Images of lung sections from virus-infected mice treated with 3D8 scFv. Lung tissues were stained with H&E. Scale bars represent 100 μm. (**E**,**F**) Viral RNA (*HA, NP*) and expression of the inflammatory factor, *IFN-β*, were analyzed by qRT-PCR. The relative concentrations of RNA were calculated after normalization to *GAPDH* expression using the delta–delta Ct method. Data are shown as the mean ± S.E.M. of triplicate samples. (**G**) Comparison of the virus titers in the lungs by a plaque assay. (**H**) Reduction in viral protein (HA) in infected lungs detected using immunohistochemistry. Nuclei were detected using DAPI (blue), and viral HA was visualized with TRITC (red). Scale bars represent 50 μm. (**I**) The mean number of HA-positive signals in the mice’s lungs. Error bars indicate SE. Asterisks indicate a significant difference determined by *t*-tests (* *p* < 0.05, ** *p* < 0.01, *** *p* < 0.001).

Lung tissue samples were collected at 5 dpi of the H1N1/H275Y challenge (Figure 3A,B). Gross lesions were present predominantly in the middle, caudal, and accessory lobes. In terms of their gross pathology, lungs from the mice in the 3D8 scFv-pretreatment group did not show any differences from the mice lungs in the uninfected WT group. Marked pulmonary congestion and tan-yellow consolidation were observed in the PBS-pretreatment and Oseltamivir-treatment groups (Figure 4A). In the intergroup comparison, the 3D8 scFv-pre- and posttreatment groups had significantly lower lung lesion scores compared with the PBS and Oseltamivir groups (*p* < 0.05). Between the 3D8 scFv-treatment groups, the gross lung lesion scores were significantly lower in the 3D8 scFv-pretreatment group than those in the 3D8 scFv-posttreatment group (*p* < 0.05). The Oseltamivir group had significantly lower gross lung lesion scores than the PBS-posttreatment groups (*p* < 0.05) (Figure 4B).

Microscopical lesions were characterized by thickened alveolar septa with increased numbers of interstitial macrophages and lymphocytes in the PBS- and Oseltamivir-treatment groups. The lungs of the 3D8 scFv-pretreatment and WT groups were normal (Figure 4C). In the intergroup comparison, the 3D8 scFv-pretreatment group had significantly lower microscopic lesion scores than the other groups (*p* < 0.01), and the 3D8 scFv-posttreatment group had significantly lower microscopic lesion scores than the PBS and Oseltamivir groups (*p* < 0.05). Microscopic lung lesion scores were not significantly different among the PBS and Oseltamivir groups (Figure 4D).

Consistently, the expression of viral RNAs and interferon beta (*IFN-β*) after viral infection was diminished in the mice lungs in the 3D8 scFv-pretreatment group compared with the mice’s lungs in the other groups, as demonstrated by the reduced expression of *HA* (68%), *NP* (71%), and *IFN-β* (51%) in the mice lungs in the 3D8 scFv-pretreatment group compared with those of the mice in the PBS-pretreatment group (Figure 4E). Moreover, the virus titer in the mice’s lungs was reduced to 10% in the 3D8 scFv-pretreatment group, compared with the PBS-pretreatment group (Figure 4G). Consistently, the signal of viral HA protein was reduced in the alveolar epithelial cell lining (Figure 4H), and the number of HA positive signals was significantly lower in the 3D8 scFv-pretreatment group compared with the Oseltamivir group (*p* < 0.05; Figure 4I).

In the 3D8 scFv-posttreatment group, the expression of viral RNA decreased to 35% (*HA*) and 18% (*NP*) (Figure 4F). Moreover, the virus titer in the mice’s lungs was reduced to 30% in the 3D8 scFv-posttreatment group compared with the virus titer in the mice’s lungs of the PBS-posttreatment group (Figure 4G). These results suggested that 3D8 scFv benefits virus-infected hosts prophylactically as well as therapeutically by reducing the viral burden and by alleviating excessive inflammation and potential further pathogenesis.

## 4. Discussion

Antigenic shifts and/or drifts are typical evolving strategies often selected by IAV, and these strategies have been major obstacles to overcome in the development of antiviral therapeutics [35,36]. The demand for pan-antiviral therapeutics that can exert a common effect regardless of IAV’s genetic mutations is increasing [37]. Oseltamivir, an unrivaled antiviral treatment against IAV, is involved in the release of viral progeny [38]. However, the H275Y missense mutation on the NA gene in the H1N1/pdm09 strain allosterically changes its binding affinity to Oseltamivir, resulting in the strain being 1500-fold less sensitive to Oseltamivir [39,40,41].

In this study, we demonstrated that 3D8 scFv has antiviral effects for multiple influenza A viruses, including Oseltamivir-resistant (H1N1/H275Y) IAV that harbors a mutation and two Oseltamivir-sensitive (H1N1/PR8 and H3N2/X-31) IAV strains. This antiviral effect was observed in both in vitro and in vivo challenges by 3D8 scFv’s intrinsic sequence nonspecific hydrolyzing activity on all types of influenza viral RNAs during the replication cycles. While 3D8 scFv pretreatment showed a prophylactic effect, 3D8 scFv posttreatment revealed the drug’s therapeutic effect (Figure S3). From the results of the in vitro influenza antiviral experiment with 3D8 scFv, we observed that 3D8 scFv increased cell viability and both reduced the expression of the viral RNA (*HA* and *NP*) and the HA protein’s signal compared with the untreated control (Figure 1). We found that the effectiveness of 3D8 scFv pretreatments had no significant difference from 3D8 scFv posttreatments in viral-infected cells, which may be because 3D8 scFv can penetrate and remain in the cytoplasm of the host cell even up to 24 hpi without any further translocation. Compared with what we observed at 2 h post infection, it is evident that the intensity of Alexa488-labeled intracellular 3D8 scFv protein increased and reached a peak after 24 h post infection (Figure 2H right panel and Figure S8B) and began to decrease gradually over time, yet still accumulated in the cytosol until 24 hpi (s H left panel and A). In other words, with 3D8 scFv pretreatment after 24 h, 3D8 scFv presented in the host cell’s cytosol upon entry of the influenza, and 3D8 scFv was degraded while the virus replicated. In contrast, with the 3D8 scFv posttreatment, 3D8 scFv gradually accumulated in the cytosol during the viruses’ life cycles (Figure 2H and Figure S8). Indeed, the amount of 3D8 scFv localized in the cytosol strongly correlated with the reduction of influenza’s genome at different time points after the viral infection (Figure 2).

**Figure 5:**
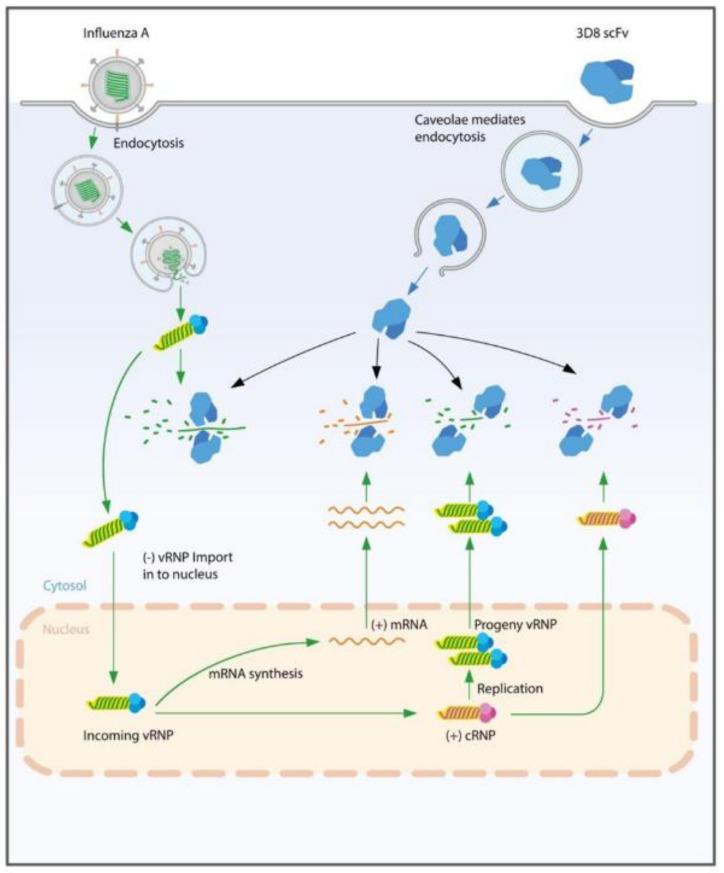
Proposed model of the antiviral mechanism of action of 3D8 scFv. 3D8 scFv, which is localized by cavolae-mediated endocytosis in the cell’s cytoplasm, does not inhibit one specific step in the virus’s life cycle. Three different viral RNAs could be targets of 3D8 scFv in all stages of IAV’s life cycle. 3D8 scFv could hydrolyze all types of IAV RNAs/RNP at different infection stages such as viral entry, viral protein biosynthesis, and virus exit in the cytoplasm of infected cells.

In general, the infection time frame of IAV may vary among IAV strains and cell types. IAVs can deliver their vRNPs from the cell surface to the nucleus in about one hour [42]. vRNAs are the original RNAs in the virion, and, after infecting cells, vRNA acts as a template for mRNA transcription to synthesize all viral proteins, including the protein for RNPs’ formation. vRNA is also a template for cRNA replication, and the progeny vRNA is only generated from the cRNA template. vRNA and cRNA are wrapped around the NP protein and a complex of polymerase proteins, but mRNA is not [11,43]. mRNA, vRNA, and cRNA can be discriminated by strand-specific real-time PCR [31,32,44]. Viral RNA replication starts in the nucleus, and then the viral infection cycle proceeds in both the nucleus and cytosol. Early studies suggested that there are different kinetics of intracellular mRNA, vRNA, and cRNA replication and suggested that vRNP and cRNP are both present in the nucleus and cytosol [44]. In our study, in mock-treated control cells, vRNAs, cRNAs, and mRNAs of H1N1/H275Y were present at different levels at an early infection stage (2–4 hpi). vRNA levels were higher than those of cRNA and mRNA because the initial vRNA level was caused by the virus inoculum itself in the cytosol, and cRNA and mRNA were later produced by vRNA in the nucleus. As time progressed after viral inoculation, cRNA, vRNA, and mRNA were gradually synthesized in the nucleus without the hydrolysis activity of 3D8 scFv. Finally, the levels of vRNA, cRNA, and mRNA increased about 10^3^–10^5^-fold at 24 hpi (Figure S10A). The localization of the three types of viral RNA in both the nucleus and cytosol were confirmed by cell fraction analysis. At 24 hpi, in the cytosol, the levels of vRNA and cRNA were relatively higher than their levels in the nucleus, but mRNA was relatively more localized in the nucleus (Figure S10B). The subcellular fractionations of nuclear and cytoplasmic fractions were qualified by Western blot analysis using a cytosolic marker (tubulin), a nuclear marker (lamin A/C), and influenza protein (nucleoprotein) (Figure S10C). The profiles of the three viral RNAs (cRNA, vRNA, and mRNA) were different in the 3D8 scFv pretreated and posttreated cells. Figure 2B–G presents quite different patterns of viral gene expression because of the different methods of 3D8 scFv treatment. In 3D8 scFv-pretreated cells, 3D8 scFv was present in the cytosol before the viral infection (Figure S8A); therefore, 3D8 scFv was able to hydrolyze the vRNA of vRNP immediately when the virus was released into the cytosol (2 hpi). As time progressed, however, the levels of 3D8 scFv slowly decreased in cytosol and virus started replication and transcription, which made the amounts of the three types of viral RNA increase gradually (Figure 2B–D). By comparison, in 3D8 scFv-posttreated cells, vRNP may have escaped from 3D8 scFv in the cytosol and entered into the nucleus for viral multiplication. 3D8 scFv can penetrate into cells via a mechanism of caveolae-mediated endocytosis [25], but 3D8 scFv cannot enter into the nucleus. Therefore, initially, the amount of all the types of viral RNAs were similar to the positive control. However, after 3D8 scFv had fully penetrated into the cytosol six hours after treatment (Figure 2 and Figure S8B), the amount of the vRNA, mRNA, and cRNA decreased because of 3D8 scFv’s RNA hydrolysis activity (Figure 2E–G). Additionally, treatment with 0.1% Triton X100 released vRNPs complex from virus particles, which were degraded by 3D8 scFv (Figure 2A). Thus, we suggested that 3D8 scFv is able to hydrolyze RNA in RNP form. Taken together, we hypothesized a mechanism of action of 3D8 scFv (Figure 5) in which, during viral infection and replication in the host cell, 3D8 scFv targets mRNA, vRNA, and cRNA equally and acts, depending on its concentration and localization in the cytoplasm, from the early to late stage of the viral cycle. It is supposed that 3D8 scFv’s antiviral activity is active throughout the entire viral cycle. Therefore, we proposed that if 3D8 scFv is administered continuously in order to maintain a constant concentration, 3D8 scFv is capable of controlling viral infections.

Consistently, in vivo intranasal administration of 3D8 scFv following viral infection revealed an improved survival rate and reduced body weight loss (Figure 3). Viral infection after 3D8 scFv pretreatment led to an increase in the survival rate from 37.5% and 30% in the PBS-pretreatment and Oseltamivir groups, respectively, to 90% in the 3D8 scFv-prettreated group. In all the tested groups, the body weight decreased daily up to 7 dpi, but only mice in the 3D8 scFv-pretreated group gradually recovered their body weight after 7 dpi (Figure 3C,D). 3D8 scFv-posttreated groups also showed 10% and 40% higher survival rates than the Oseltamivir- and PBS-posttreated groups, respectively. We investigated the therapeutic effect of 3D8 scFv treatment after viral infection to evaluate its potential as a drug that could be administered intranasally upon the initiation and development of viral pathogenesis. Intranasal administration of PBS, which served as a control, after viral infection resulted in a survival rate of 0% at 8 dpi (Figure 3E). However, the survival rate in the 3D8 scFv-posttreatment group was 40%, and body weight was restored after 10 dpi (Figure 3F). Comparison of the clinical symptoms revealed that 3D8 scF-posttreatment resulted in 3D8 scFv’s observable antiviral effects compared with the PBS-posttreatment group (Figure 4). Following viral infection, even when administered intranasally, 3D8 scFv acts as an effective therapeutic drug.

The difference in the survival rates and body weight loss between the 3D8 scFv-pretreated and 3D8 scFv-posttreated groups were attributable mainly to 3D8 scFv’s presence in infected cells and tissues as well as the side effects of anesthesia after viral infection. Based on the in vitro virus challenging experiments, the presence of 3D8 scFv in the cells in advance of the viral infection was critically important to control the virus’s proliferation as viral multiplication rates were higher than 3D8 scFv’s rate of penetration. In addition, the side effects of anesthesia weakened the mice. To treat 3D8 scFv intranasally, the mice had to be anesthetized for every procedure.

Comparative assessment of the clinical indications (lung morphology, lung tissue sections, viral RNA/protein/inflammation factors, and number of plaques) between the treatment groups revealed that 3D8 scFv pretreatment showed a strong antiviral effect with minimal cytotoxicity compared with PBS pretreatment or Oseltamivir treatment (Figure 4). This is because, over a time frame of 48 h, 3D8 scFv was localized in the epithelial lining of the bronchioles/alveoli such that when the cells were subsequently infected with the virus, the preexisting 3D8 scFv started to mediate its antiviral effects during the early stages of the infection (Figure 4 and Figure S9). Based on this, 3D8 scFv has the potential to be used as a prophylactic drug for influenza.

We previously reported that the presence of 3D8 scFv in epithelial cells and the lung alveoli exhibited the preventive activity against IAV [26]. In this study, we confirmed that when 3D8 scFv is administered intranasally (IN), it possibly passes through the respiratory surface and penetrates the epithelial cells of the bronchioles/alveoli (Figure S9D). There are several methods for drug delivery, such as IN, intraperitoneal (IP), and intravenous (IV) deliveries and oral administration (OA), for injecting substances into in vivo models. Because 3D8 scFv is a protein, OA is inappropriate because it can be digested in the digestive system. Therefore, IN, IP, and IV deliveries have a potential for drug administration of substances below 40 kDa; however IP or IV administration through blood vessels means that delivered drugs are discharged from the body within a short period of time thanks to the kidneys’ efficient renal clearance [45]. Based on the characteristics of 3D8 scFv and respiratory IAV, we used IN administration for the delivery of 3D8 scFv because IN administration can directly deliver 3D8 scFv to the lungs, where this drug can be stably contained [46]. The mucosal surface is a selectively permeable barrier that covers the surface of internal organs and prevents the passage of toxins and bacteria [47,48]. The mucosal layer covers the respiratory (nose, trachea, and lungs) and digestive (intestine) epithelial cells. For the body to absorb a drug, it must pass through these layers [49]. The above-mentioned results imply that 3D8 scFv can potentially be used to manage infections of organs with a mucosal layer, including the respiratory tract. Given that 3D8 scFv is capable of passing through epithelial cells and possibly mucosal layers, 3D8 scFv may be an important candidate for treating diseases resulting from respiratory viral infections.

Because 3D8 scFv is a bio pharmaceutical therapeutic antibody, 3D8 scFv can also be used genetically depending on the expression host. In our previous studies, we showed 3D8 scFv’s antiviral effect against H9N2 and Newcastle disease using the 3D8 scFv transgenic chicken model [50,51]. Moreover, we also demonstrated 3D8 scFv’s antiviral effect against murine norovirus using in vivo administration in 3D8 scFv-expressing lactobacilli. Further systemic and careful investigation into the toxicity and potential immune-related adverse effects of 3D8 scFv treatment should be performed to evaluate and predict the clinical efficacy of 3D8 scFv as an antiviral drug for patients.

In conclusion, 3D8 scFv can be applied as a potential prophylactic as well as a therapeutic drug against WT or mutated IAVs because it directly hydrolyzes viral RNA or vRNP. Consequently, 3D8 scFv blocks viral proliferation in a nonsequence-specific RNase manner. Furthermore, 3D8 scFv can be used in new IAV outbreaks as well as with emerging known or unknown viruses as the time-consuming process of viral identification and characterization are not necessary to develop new therapeutic antiviral drugs. To the best of our knowledge, there are presently no reports of influenza drugs that target viral RNA. Therefore, with new viral RNA targets, antiviral drugs such as 3D8 scFv can be developed to combat influenza viruses and even other hard-to-control, novel viruses such as COVID-19. 

## Data Availability

Not applicable.

## Supplementary Materials

The following supporting information can be downloaded at: https://www.mdpi.com/article/10.3390/v14051105/s1: Figure S1, Antiviral activity of Oseltamivir against the three influenza virus strains. (A) Viability of virus-infected MDCK cells following Oseltamivir treatment. MDCK cells were infected with viruses (MOI = 0.1) for 1 h followed by the removal of the viral inoculum. The medium was replaced with new media containing various concentrations of Oseltamivir (serially two-fold dilution in PBS with a starting concentration of 500 μM). After incubation for 48 h at 37 °C, cell viability was measured using an MTT assay. Data are shown as the mean ± S.E.M. of triplicate samples. Figure S2, Cytotoxicity of 3D8 scFv in A549 cells. (A) A549 cells were incubated in the presence of various concentrations of 3D8 scFv (0–40 μM) for 48 h at 37 °C. Cell viability was measured using an MTT assay. (B) Identification of DEGs through transcriptomic analysis in A549 cells. Volcano plots were used to compare DEGs (FDR < 0.05 and log_2_ FC ≥ 1; Up, log_2_ FC ≤ −1; Down) at dosages of 3D8 scFv (5, 10, and 40 μM). The *x*- and *y*-axes of the volcano plots were scaled by log_2_-fold changes and -log_10_  *p*-values. Figure S3, Schematic diagram for testing the antiviral effect of 3D8 scFv pre/posttreatment against IAV. Figure S4, Dose-dependent antiviral activity of 3D8 scFv against H1N1/PR8 and H3N2/X-31 strains. (A,B) The relative viability of 3D8 scFv-pretreated MDCK cells against viruses. 3D8 scFv treatment was conducted following the scheme illustrated in Figure S3. Cell viability was measured using an MTT assay. (C,D) The relative viability of 3D8 scFv-posttreated MDCK cells against viruses. Cell viability was measured using an MTT assay. Data are shown as the mean ± S.E.M. of triplicate samples. Error bars indicate standard error (SE). Asterisks indicate significant differences determined by the *t*-test (* *p* < 0.05, ** *p* < 0.01, *** *p* < 0.001). Figure S5, Inhibition of H1N1/PR8 and H3N2/X-31 IAV proliferation by 3D8 scFv. (A,C) The relative viral RNA expression (*HA, NP*) in 3D8 scFv pretreated MDCK cells. 3D8 scFv treatment was conducted following the scheme illustrated in Figure S3. qRT-PCR was performed to measure the relative expression of viral RNAs. (B,D) The relative viral RNA expression in 3D8 scFv-posttreated MDCK cells. qRT-PCR was performed to measure the relative expression of viral RNAs. Data are shown as the mean ± S.E.M. of triplicate samples. Error bars indicate SE. * *p* < 0.05, ** *p* < 0.01, *** *p* < 0.001. Figure S6, Reduction of H1N1/PR8 viral HA protein by 3D8 scFv treatment. The detection of viral protein (HA) in infected MDCK cells using immunocytochemistry at 24 hpi. (A) 3D8 scFv pretreatment. (B) 3D8 scFv posttreatment. 3D8 scFv treatment and viral infection were the same as described in the previous section. Nuclei were detected using DAPI (blue), 3D8 scFv with Alexa Fluor 488 (green), and viral HA with TRITC (red). Scale bars represent 20 μm. Figure S7, Comparison of changes in viral *NP* vRNA, cRNA, and mRNA by 3D8 scFv treatment. (A–C) The relative viral *NP* vRNA, cRNA, and mRNA expression in 3D8 scFv-pretreated MDCK cells. MDCK cells were incubated with 3D8 scFv (3 μM), infected with viruses (MOI = 0.1), and qRT-PCR was performed to measure the relative expression of viral RNAs as described in the Materials and Methods section. (D–F) The relative viral *NP* vRNA, cRNA, and mRNA expression in 3D8 scFv-posttreated MDCK cells. qRT-PCR was performed to measure the relative expression of viral RNAs as described in the Materials and Methods. Data are shown as the mean ± S.E.M. of triplicate samples. Figure S8, Comparison of intracellular 3D8 scFv retention time by 3D8 scFv treatment method. (A) Pretreatment (B) Posttreatment. Changes in the amount of 3D8 scFv in the cytoplasm according to pre/posttreatment. 3D8 scFv treatment was conducted following the scheme illustrated in Figure S3. Nuclei were detected by DAPI staining (blue), and 3D8 scFv was visualized using antibodies conjugated with Alexa Fluor 488 fluorescent dye (green). Scale bars represent 20 μm. Figure S9, Evaluation of the pharmacological properties of 3D8 scFv in vivo. (A) Schematic diagram of 3D8 scFv treatment. 3D8 scFv (50 μg/d) was administered intranasally to mice for 4 d. (B) A morphological comparison of the mice’s lungs. Lungs were harvested every day for 5 d. (C) A histopathological comparison of H&E-stained lung sections from mice treated with 3D8 scFv. Scale bars represent 100 μm. (D) 3D8 scFv penetration in lung tissues was detected using immunohistochemistry. Nuclei and 3D8 scFv were detected using DAPI (blue) and Alexa Fluor 488 (green). Scale bars represent 50 μm. (E) 3D8 scFv retention time in the mice’s lungs was analyzed using Western blotting. Figure S10, Measurement of viral *HA* RNA in cells after H1N1/H275Y infection. (A) Relative viral *HA* vRNA, cRNA, and mRNA expression in H1N1/H275Y-infected MDCK cells. qRT-PCR was performed to measure the relative expression of viral RNAs. (B) Comparison of subcellular (cytosol, nucleus) viral vRNA, cRNA, and mRNA relative expression levels. qRT-PCR was performed to measure the relative expression of viral RNAs. Data are shown as the mean ± S.E.M. of triplicate samples. (C) Qualification of the subcellular fractionation by Western blot analysis using a cytosolic marker (tubulin), nuclear marker (lamin A/C), and influenza protein (Nucleoprotein). Table S1, Specific primers used in qRT-PCR. Table S2, Specific primers used in qPCR. Table S3, Specific primers used in RT-PCR (Mechanism). Table S4, Specific primers used in qPCR (Mechanism).
