# Supplementary figures and images for: A Therapeutically Active Minibody Exhibits an Antiviral Activity in Oseltamivir-Resistant Influenza-Infected Mice via Direct Hydrolysis of Viral RNAs
Source: Viruses. 2022 May 21;14(5):1105. doi: 10.3390/v14051105 (PMC9146509; doi:10.3390/v14051105)

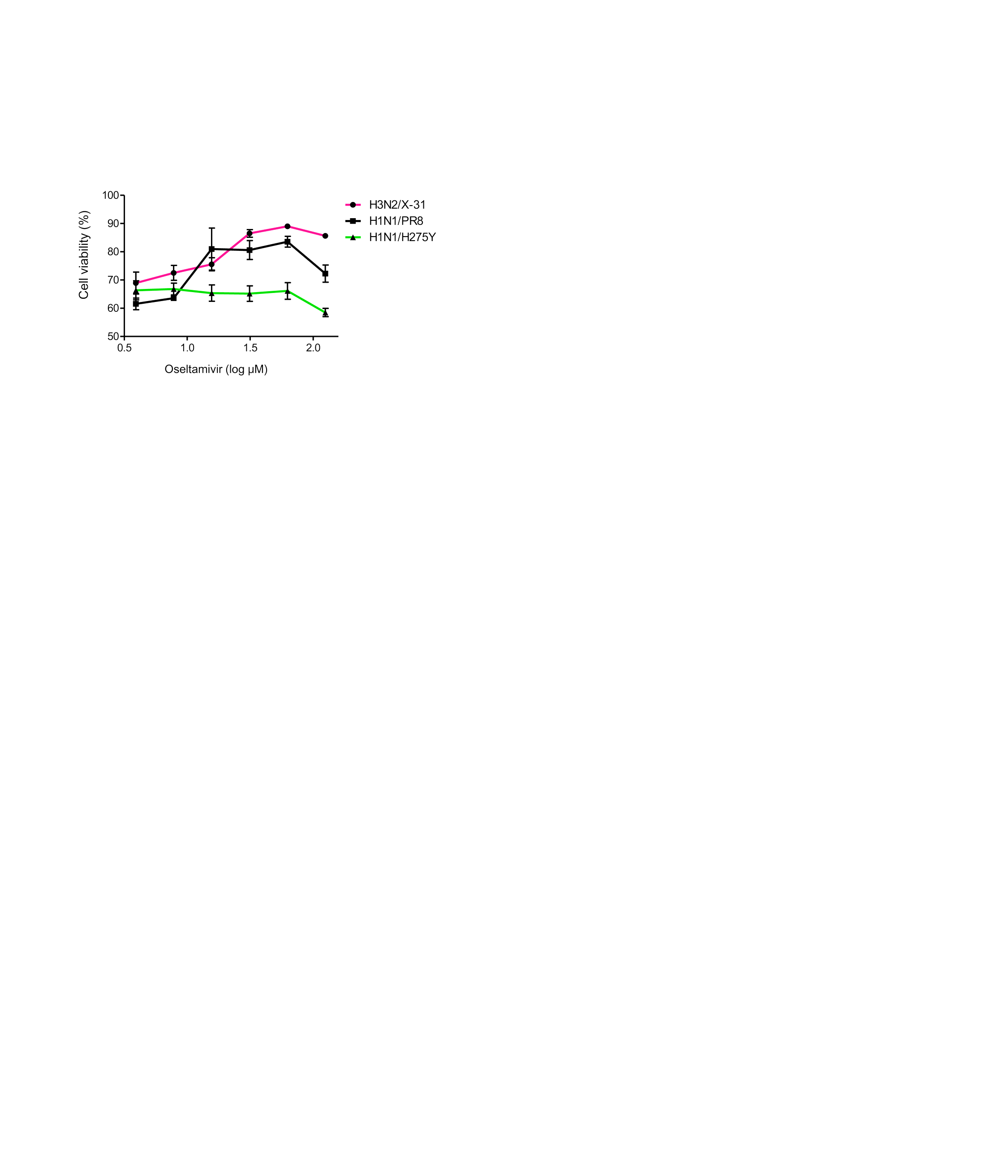

Supplement: Supplementary file 1 [file viruses-14-01105-s001.zip › S1 Fig.tif]

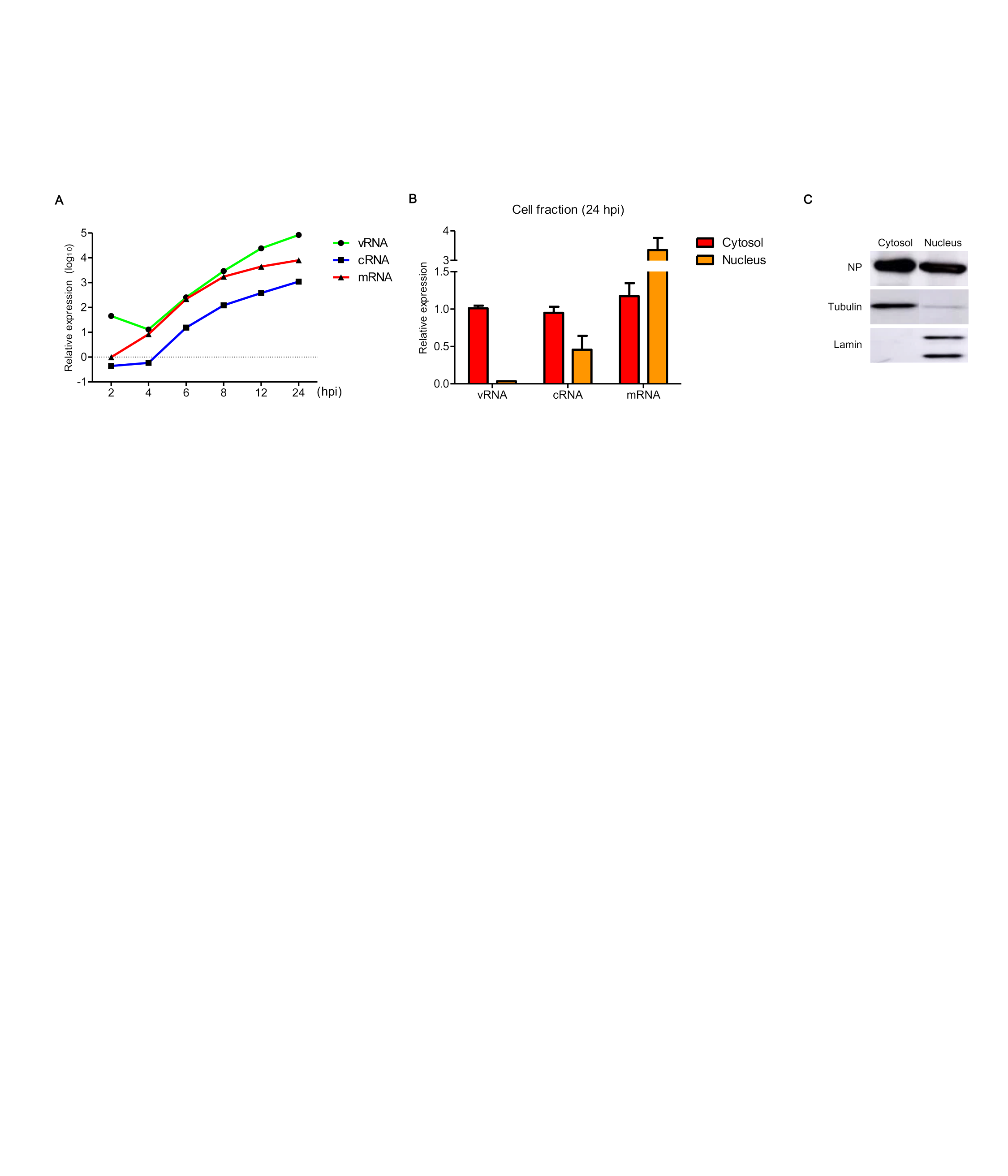

Supplement: Supplementary file 1 [file viruses-14-01105-s001.zip › S10 Fig.tif]

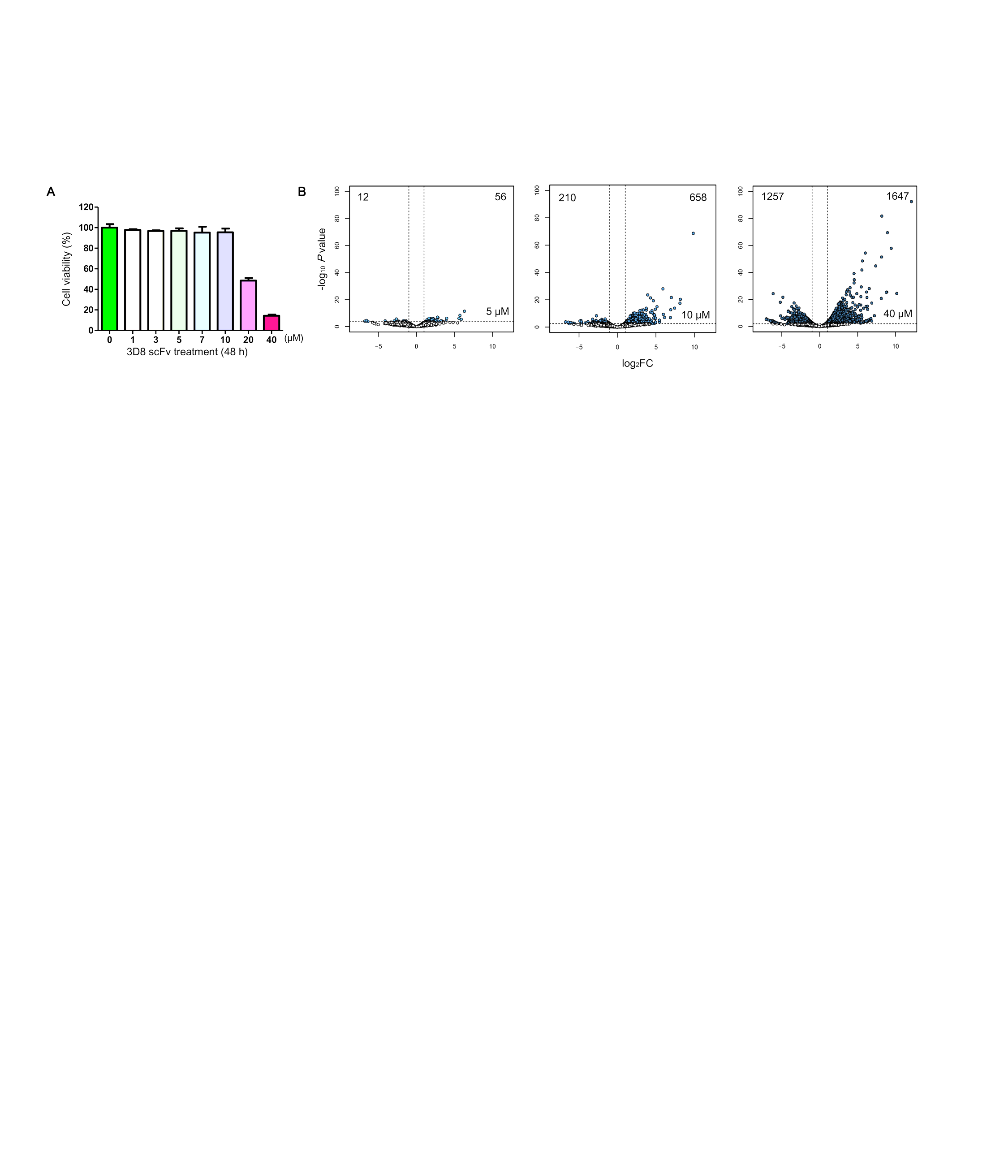

Supplement: Supplementary file 1 [file viruses-14-01105-s001.zip › S2 Fig.tif]

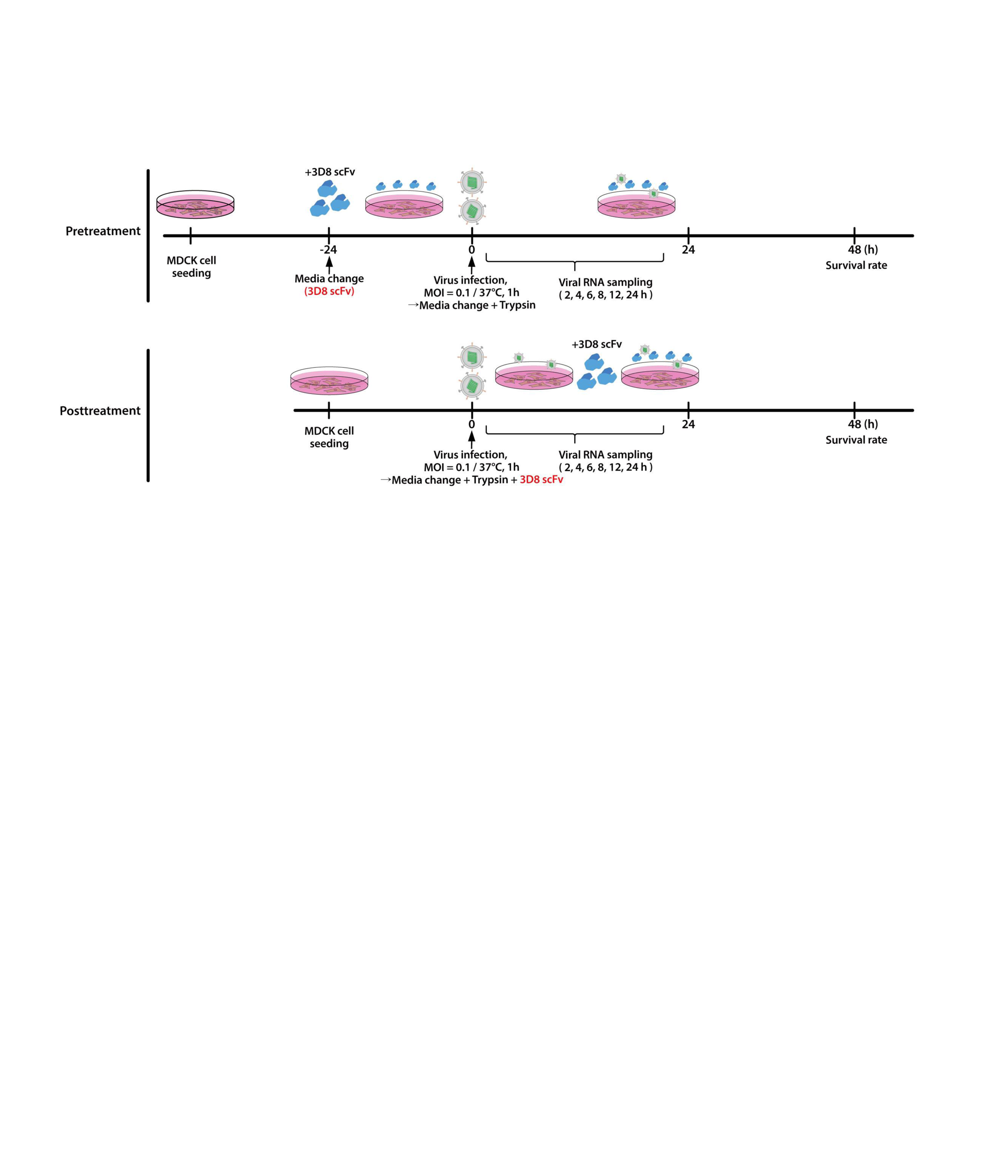

Supplement: Supplementary file 1 [file viruses-14-01105-s001.zip › S3 Fig.tif]

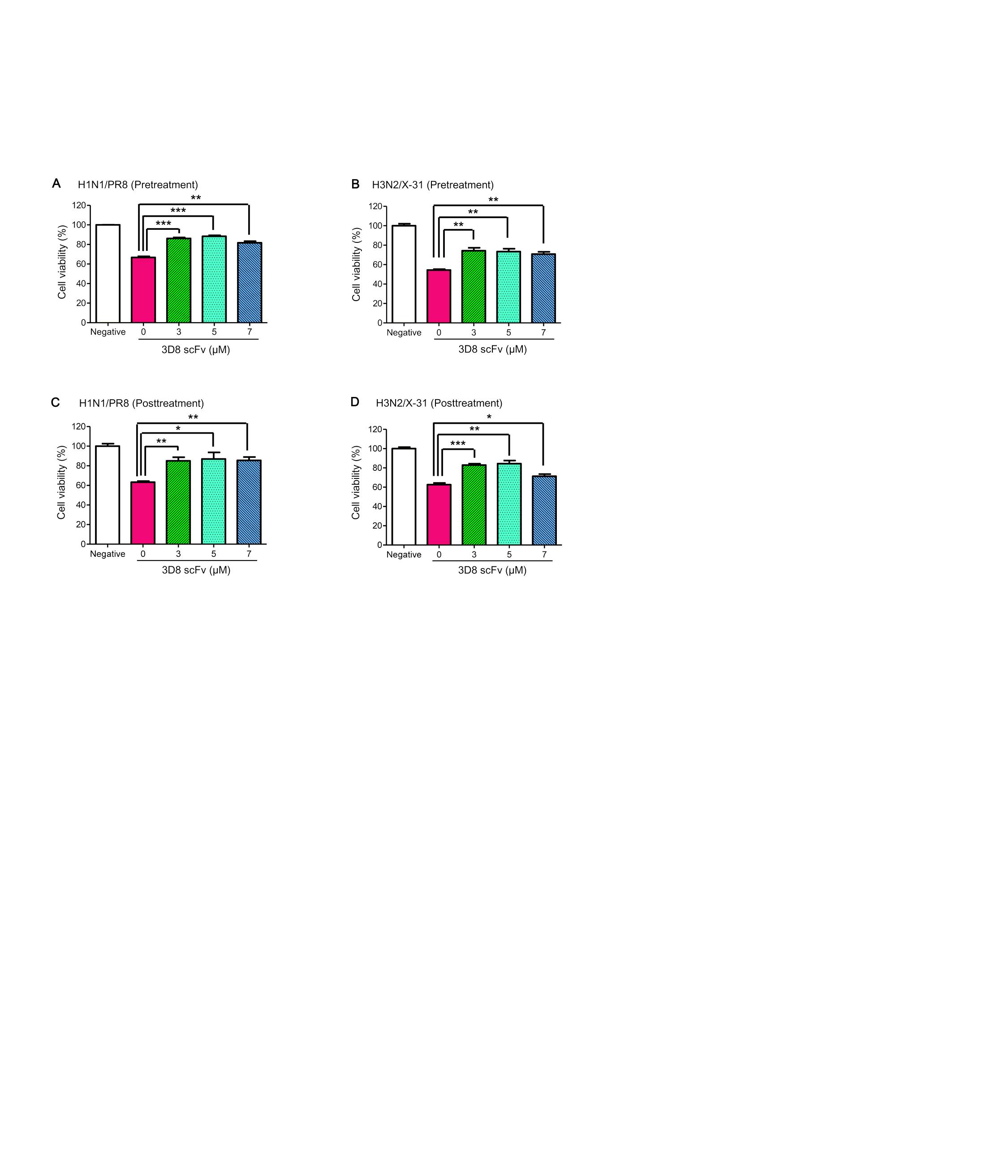

Supplement: Supplementary file 1 [file viruses-14-01105-s001.zip › S4 Fig.tif]

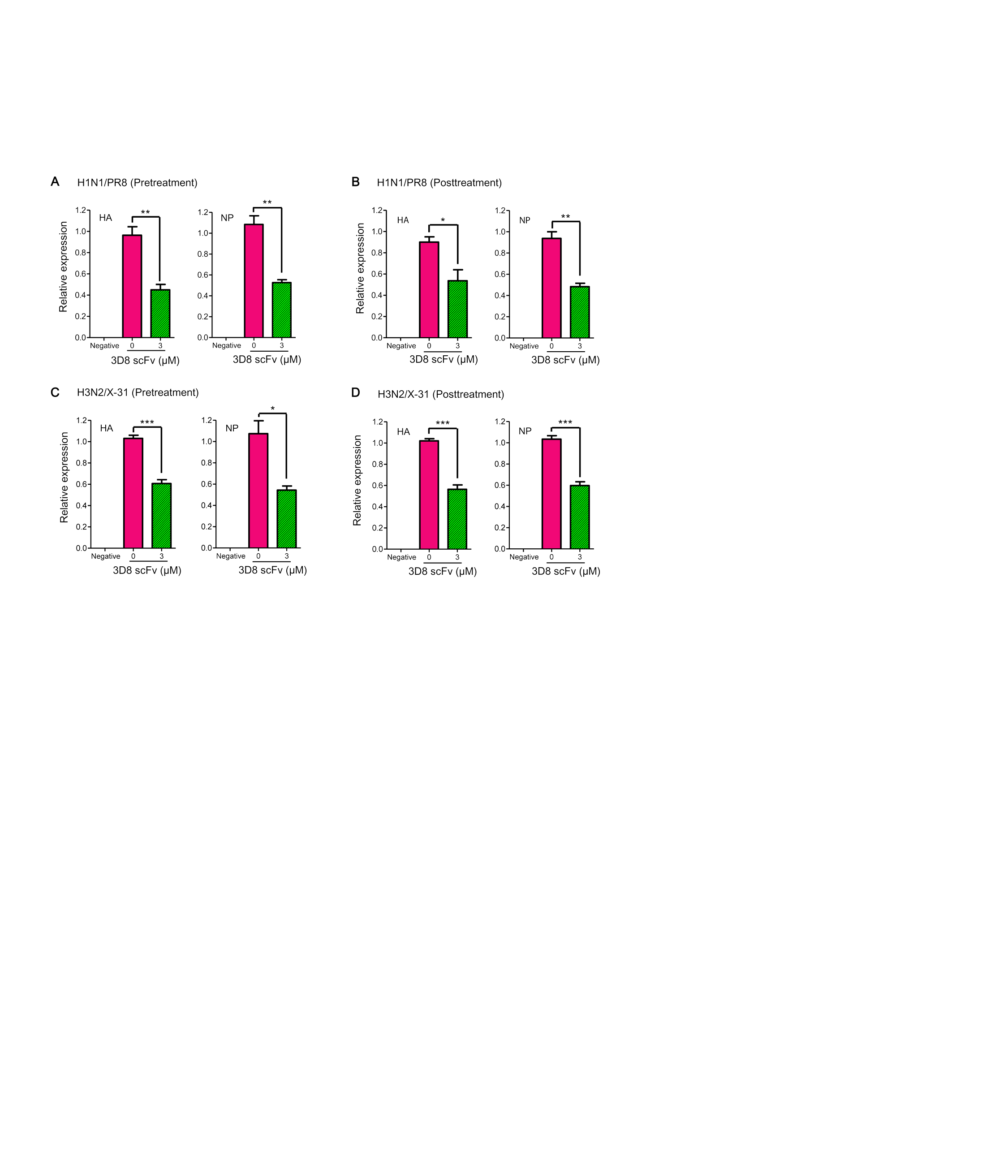

Supplement: Supplementary file 1 [file viruses-14-01105-s001.zip › S5 Fig.tif]

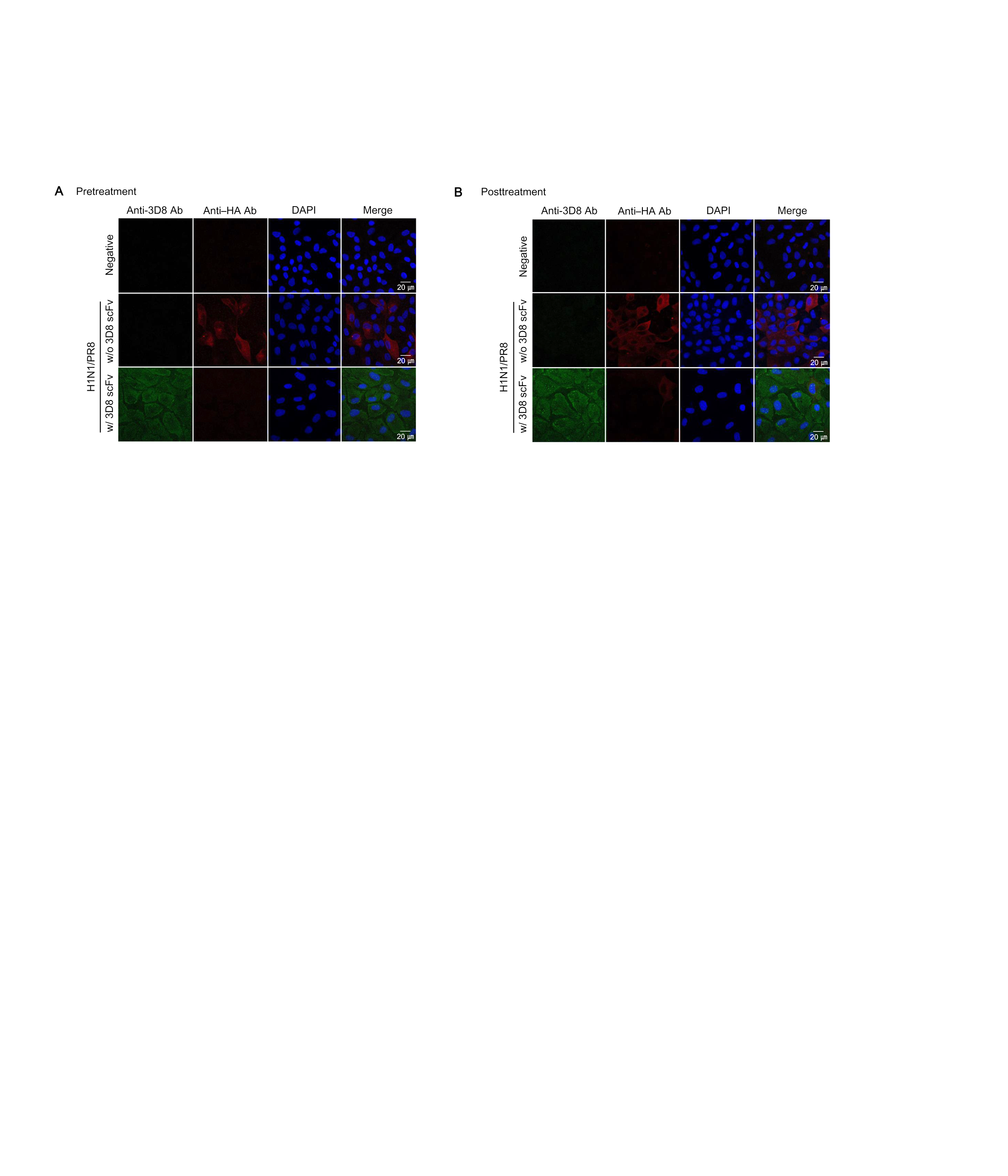

Supplement: Supplementary file 1 [file viruses-14-01105-s001.zip › S6 Fig.tif]

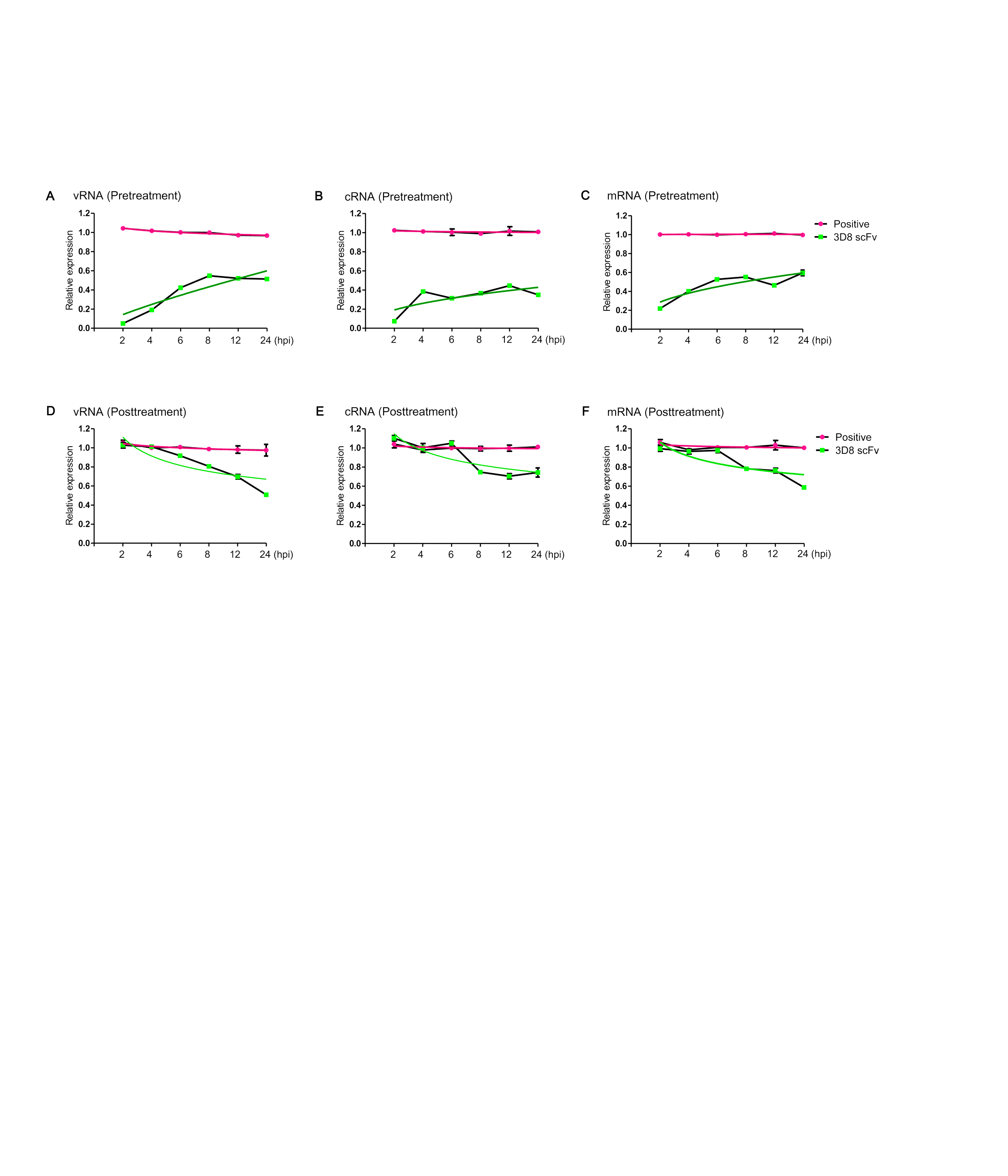

Supplement: Supplementary file 1 [file viruses-14-01105-s001.zip › S7 Fig.tif]

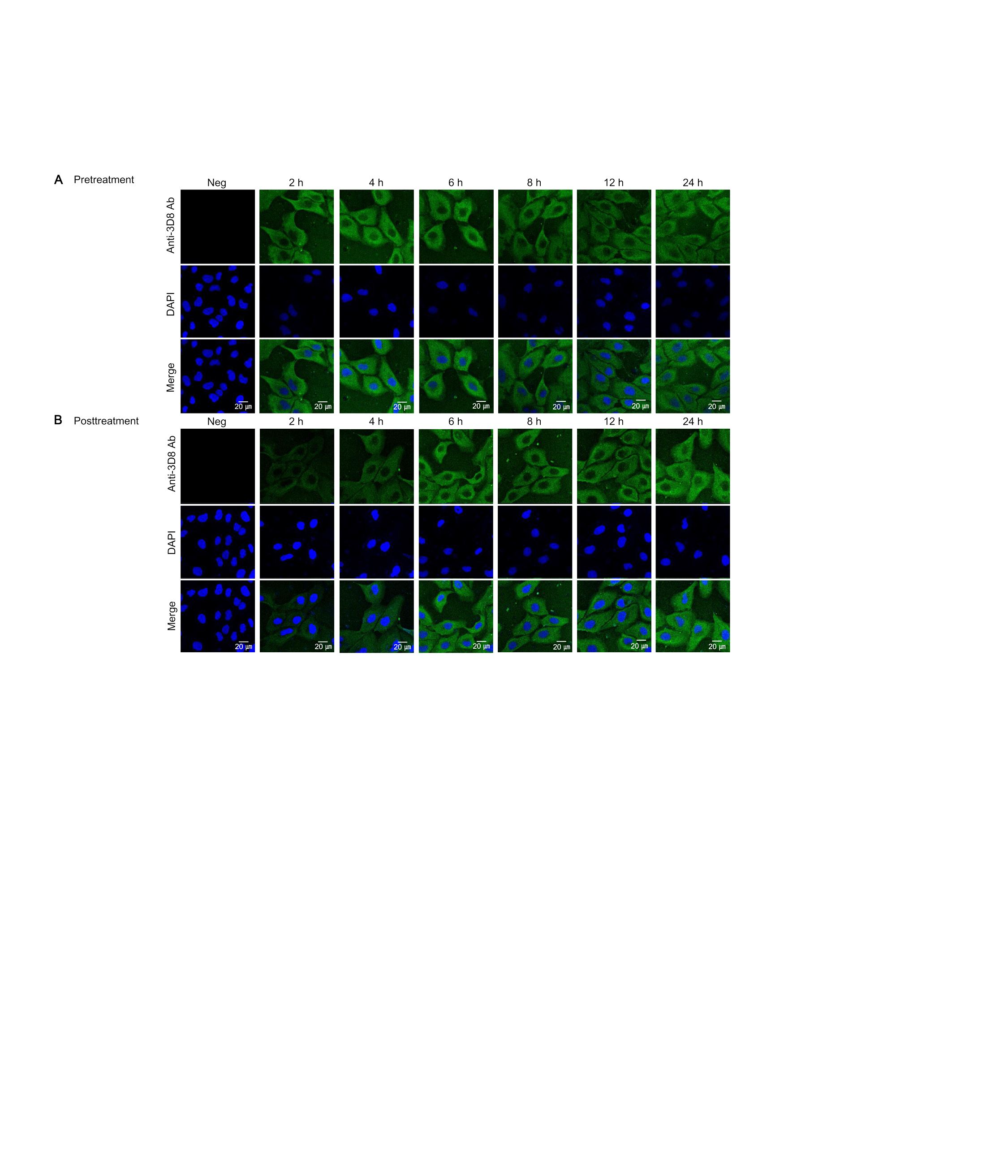

Supplement: Supplementary file 1 [file viruses-14-01105-s001.zip › S8 Fig.tif]

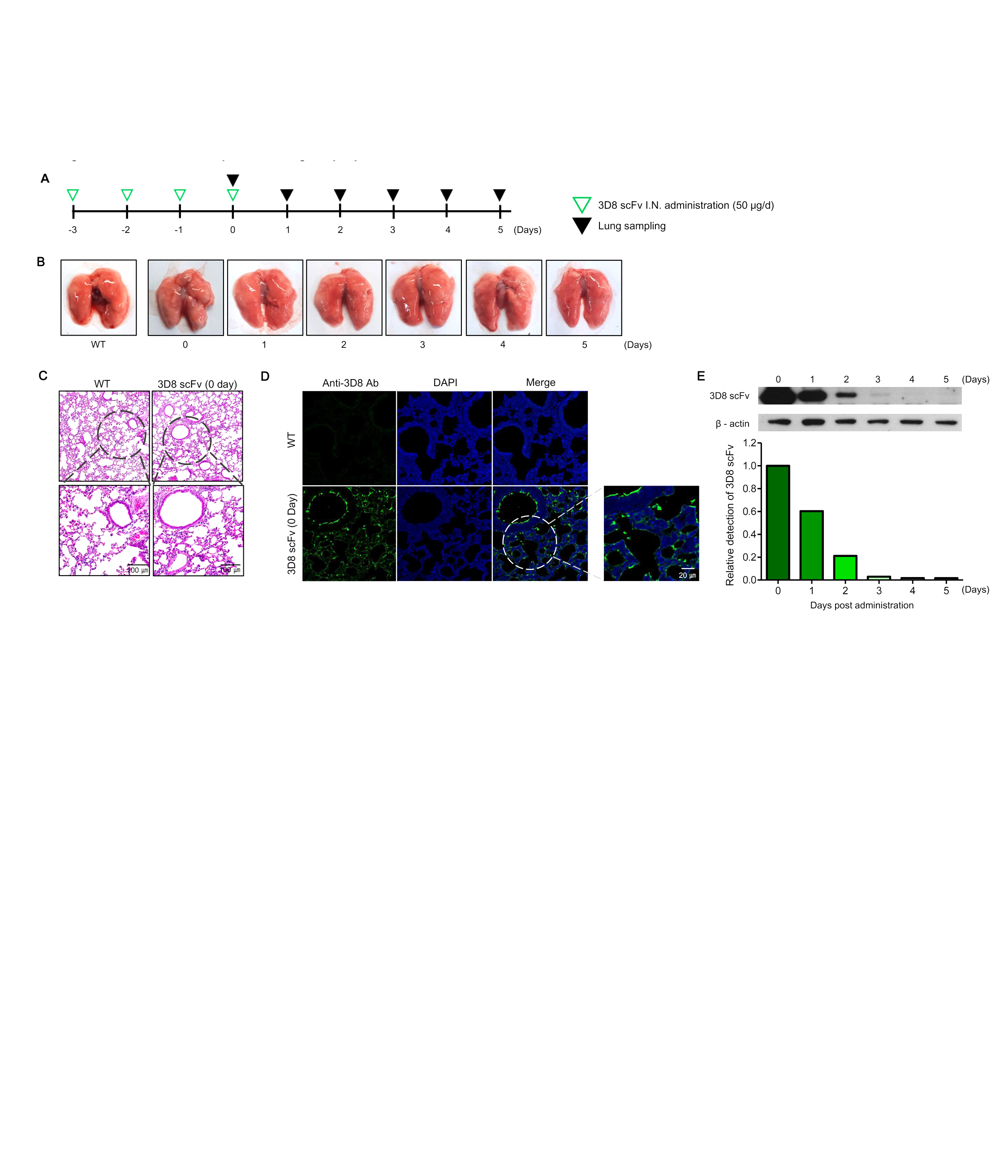

Supplement: Supplementary file 1 [file viruses-14-01105-s001.zip › S9 Fig.tif]

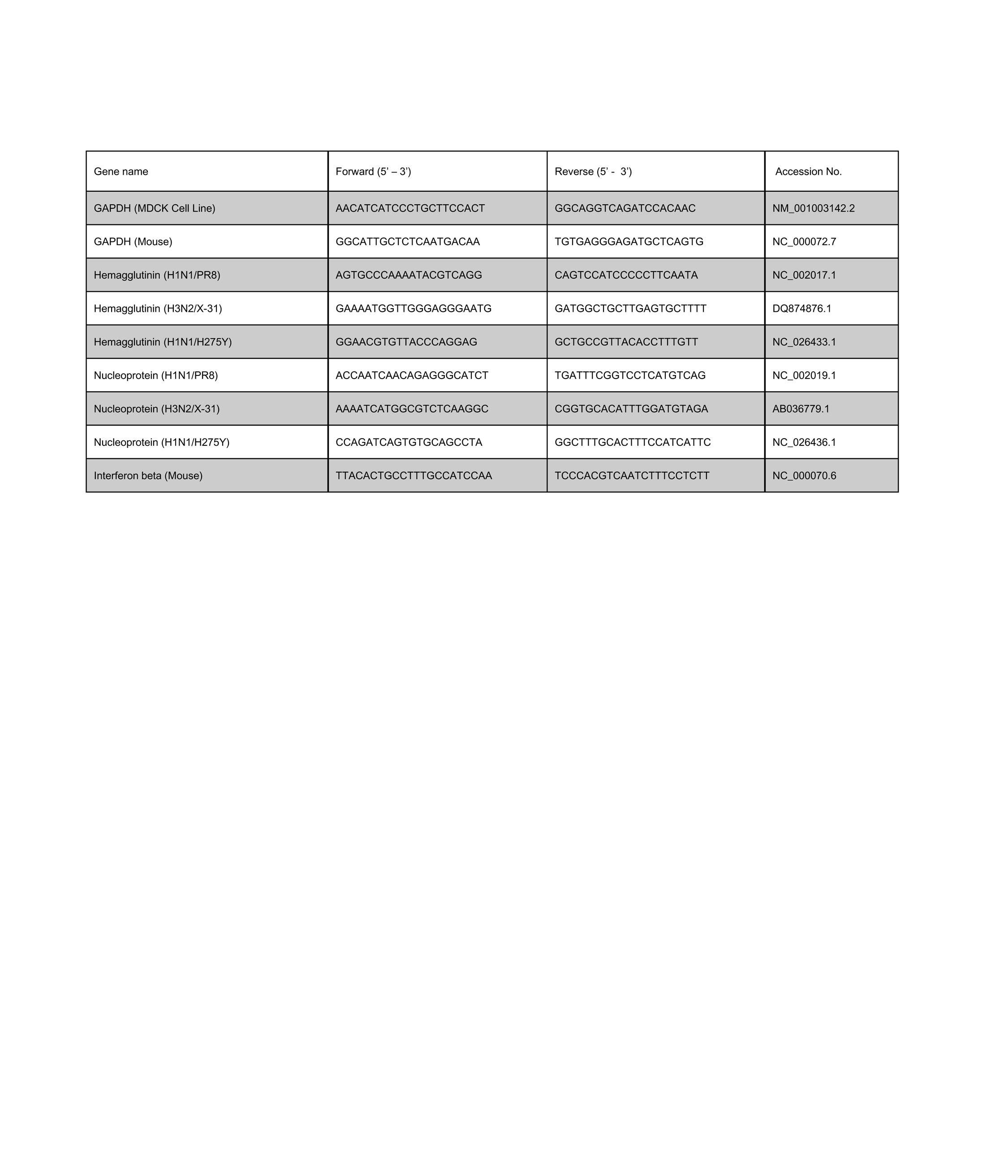

Supplement: Supplementary file 1 [file viruses-14-01105-s001.zip › Table S1.tif]

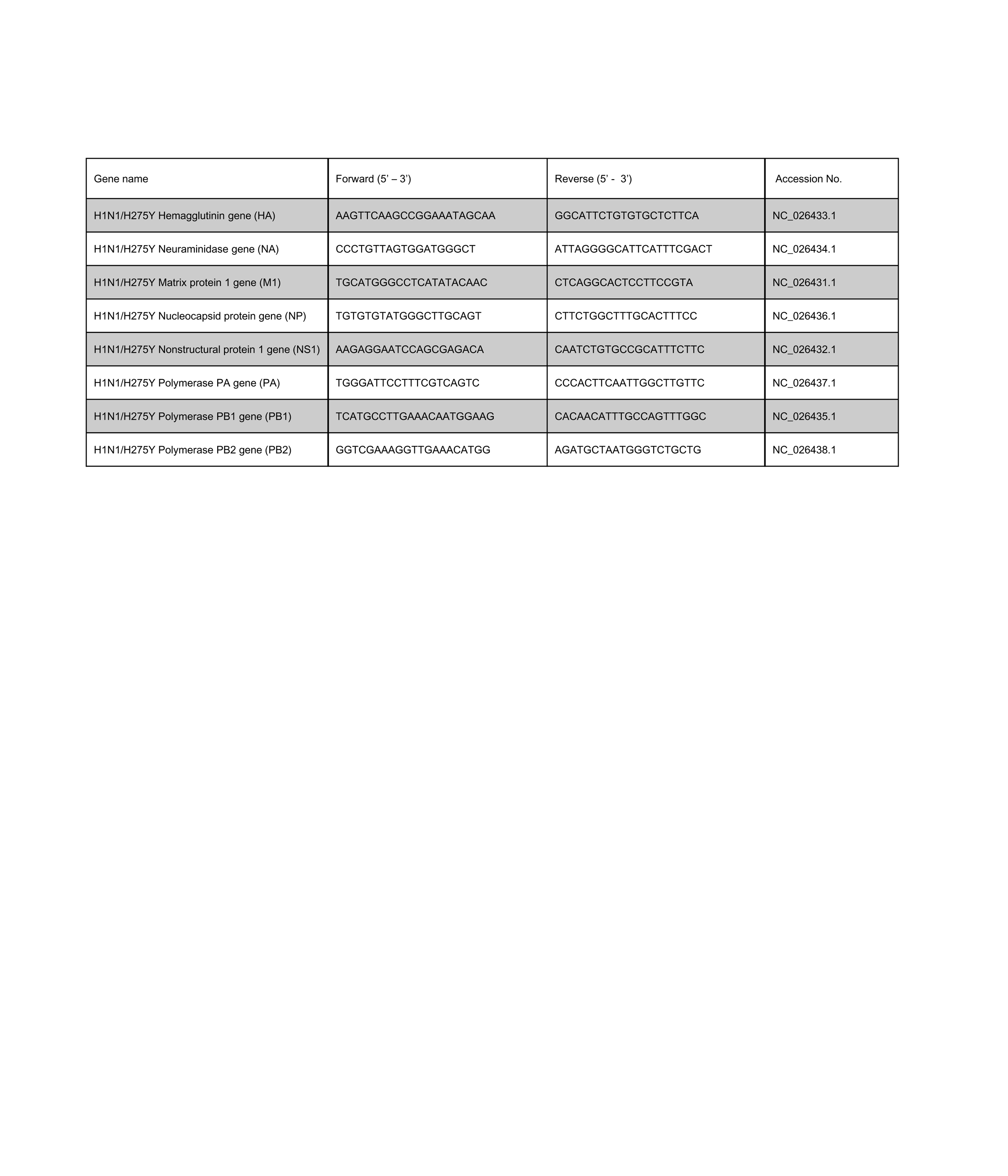

Supplement: Supplementary file 1 [file viruses-14-01105-s001.zip › Table S2.tif]

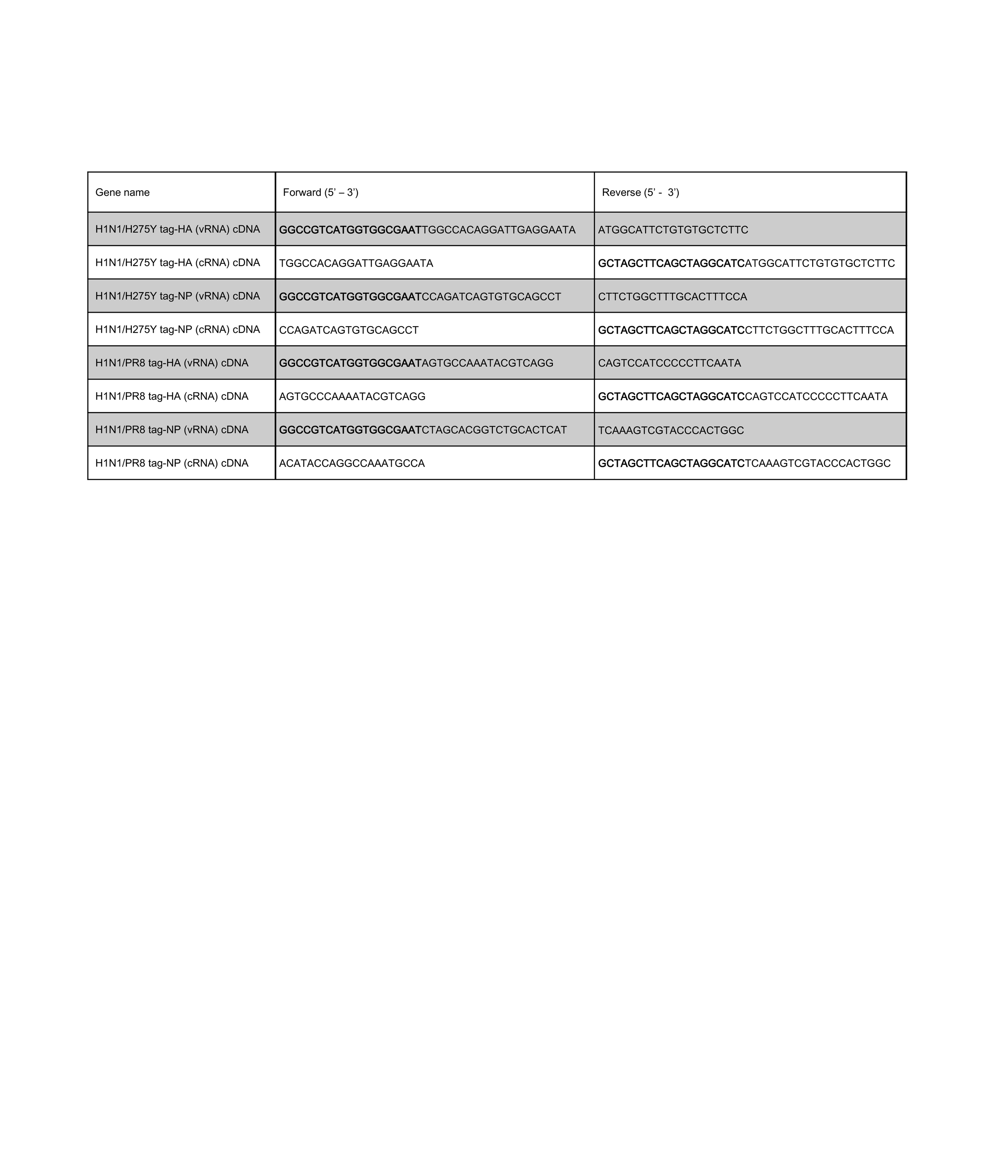

Supplement: Supplementary file 1 [file viruses-14-01105-s001.zip › Table S3.tif]

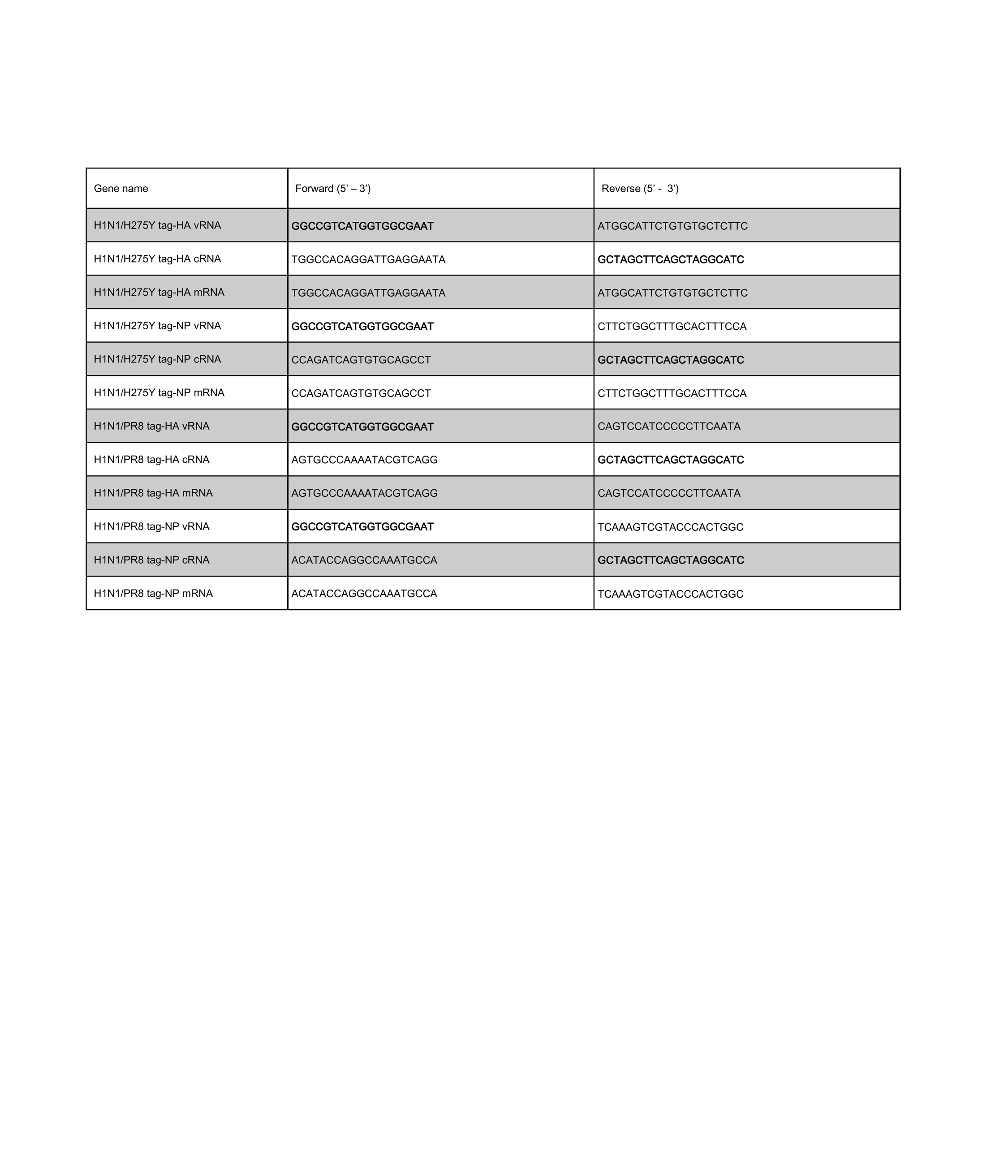

Supplement: Supplementary file 1 [file viruses-14-01105-s001.zip › Table S4.tif]
